# Ultra-deep sequencing enables high-fidelity recovery of biodiversity for bulk arthropod samples without PCR amplification

**DOI:** 10.1186/2047-217X-2-4

**Published:** 2013-03-27

**Authors:** Xin Zhou, Yiyuan Li, Shanlin Liu, Qing Yang, Xu Su, Lili Zhou, Min Tang, Ribei Fu, Jiguang Li, Quanfei Huang

**Affiliations:** 1 BGI-Shenzhen, Beishan Industrial Zone, Yantian District, Shenzhen, Guangdong Province 518083, China; 2 China National GeneBank-Shenzhen, Yantian District, Shenzhen, Guangdong Province 518083, China; 3 Shenzhen Key Laboratory of Environmental Microbial Genomics and Application, Shenzhen, Guangdong Province 518083, China

**Keywords:** Next-generation-sequencing, Species richness, Abundance, Biomonitoring, Insect biodiversity, Mitochondria, PCR-independent, Metabarcoding

## Abstract

**Background:**

Next-generation-sequencing (NGS) technologies combined with a classic DNA barcoding approach have enabled fast and credible measurement for biodiversity of mixed environmental samples. However, the PCR amplification involved in nearly all existing NGS protocols inevitably introduces taxonomic biases. In the present study, we developed new Illumina pipelines without PCR amplifications to analyze terrestrial arthropod communities.

**Results:**

Mitochondrial enrichment directly followed by Illumina shotgun sequencing, at an ultra-high sequence volume, enabled the recovery of *Cytochrome c Oxidase subunit 1* (*COI*) barcode sequences, which allowed for the estimation of species composition at high fidelity for a terrestrial insect community. With 15.5 Gbp Illumina data, approximately 97% and 92% were detected out of the 37 input Operational Taxonomic Units (OTUs), whether the reference barcode library was used or not, respectively, while only 1 novel OTU was found for the latter. Additionally, relatively strong correlation between the sequencing volume and the total biomass was observed for species from the bulk sample, suggesting a potential solution to reveal relative abundance.

**Conclusions:**

The ability of the new Illumina PCR-free pipeline for DNA metabarcoding to detect small arthropod specimens and its tendency to avoid most, if not all, false positives suggests its great potential in biodiversity-related surveillance, such as in biomonitoring programs. However, further improvement for mitochondrial enrichment is likely needed for the application of the new pipeline in analyzing arthropod communities at higher diversity.

## Background

Given the increasing needs for assessing habitat quality and conserving natural bio-resources, biodiversity composition and its temporal and spatial variations have been evaluated systematically using standardized protocols [1,2]. National biomonitoring programs have been established globally, such as in the United States, United Kingdom, Australia and Canada [3-6]. Although specific protocols and sampling scales vary across nations, tens of thousands of sampling sites are collected multiple times a year, through which millions of biological specimens are routinely collected, preserved, identified and statistically analyzed [7]. This biological information is used by environmental agencies as the scientific basis for decision-making [2,3,5,8]. However, the major impediment to this application has been the limited capacities in morphological identification of taxonomic diversity for large volumes of biological samples in an accurate and high-throughput manner [9]. Although many diversity analyses employed in biological assessments are of high-quality and credibility, the limited identification capacity may lead to coarse diversity resolution [10], inconsistent taxonomic delineation across individual researchers and institutes [11], much reduced sampling scale [7], and extended turn-around time in routine sample processes [12].

DNA barcoding, which utilizes a standard gene fragment for species identification, has been widely used to facilitate biodiversity and ecological studies [13]. When the classic DNA barcoding approach, which is optimized based on individual Sanger sequencing, is coupled with next-generation-sequencing (NGS) technologies, the combined method, *metabarcoding*[14], shows even greater potential in unveiling molecular characteristics of the entire fauna or flora in question. In particular, metabarcoding has enabled sophisticated analyses of biodiversity in varied environments, ranging from deep-sea meiofauna [15] to terrestrial insects collected by Malaise traps [16], while the majority of studies have focused on microbial communities [17,18]. In addition to the ability to reveal diversity for mixed biological samples, NGS platforms are capable of high-throughput sequencing [19,20] with short turn-around time (e.g., 24 hours for the Illumina MiSeq and Roche 454 GS FLX + sequencers).

**Figure 1:**
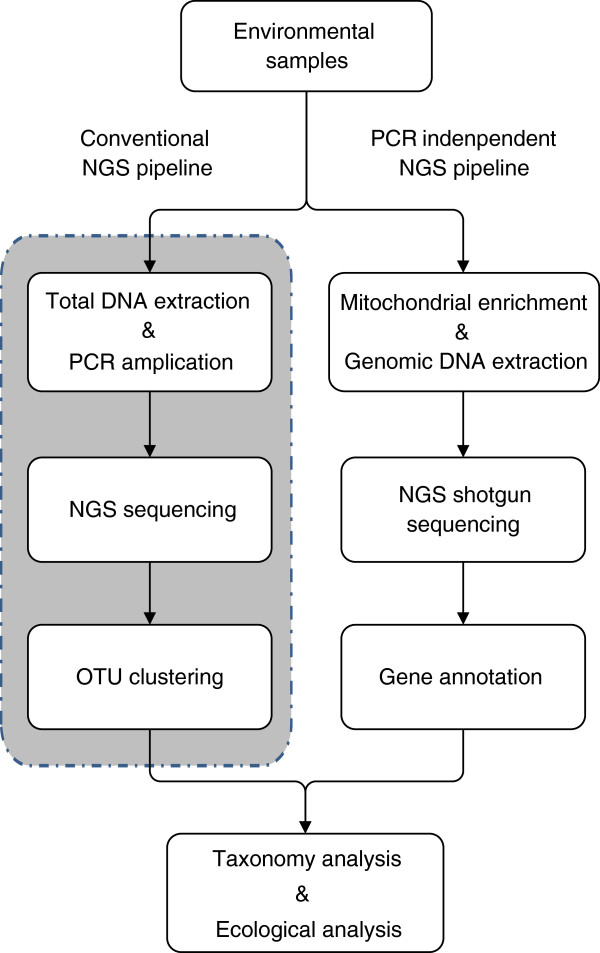
Schematic pipelines of conventional and PCR-independent NGS biodiversity analyses.

In nearly all published works, NGS analyses of biodiversity usually involve DNA extraction of bulk samples (mixtures of co-occurring taxonomic groups), PCR amplification of targeted genetic markers, and NGS analysis for taxonomic composition (Figure 1). PCR amplification of targeted genes is employed as the sole approach to acquiring sufficient barcode sequences that are used for species identification. An inherent drawback to this approach is that primers designed to amplify the full range of taxa presented in the bulk sample are rarely “universal”, with different amplification efficiencies in varied organisms [21-28]. Although much effort has been made to increase the universality of primer sets [29-32], it is difficult to predict the performance of primers when the investigated fauna is largely unknown. As a result, it seems to be impossible to completely eliminate the taxonomic biases introduced by PCR. In addition, our recent work (unpublished data) found that amplification errors (such as mismatches to the template DNA) propagated during PCR could be readily detected by the highly sensitive NGS technology, therefore increasing the ratio of false positives – which is potentially one of the major causes of what is commonly known as “biodiversity inflation” or “false positives” found in nearly all published NGS analyses of biodiversity [16,33]. Furthermore, the success of PCR amplifications is also influenced by the nucleotide composition and secondary structure of the DNA templates. For instance, homopolymers – a long strain of identical nucleotides arranged in tandem, present a challenge for the polymerase to pass through. If these nucleotide characteristics are taxon specific, amplification efficiency will create taxonomic biases despite primer optimization. The DNA barcoding of subgroups of Hymenoptera presents a notoriously difficult example where poly-Ts are commonly found in regions of the *Cytochrome c Oxidase subunit 1* gene (*COI*) barcodes. This characteristic has led to low success in the acquisition of both full-length barcodes [34,35] and in taxonomic detection for a large portion of Hymenoptera in recent NGS biodiversity analyses [16]. Lastly, once PCR is included in the NGS analytical pipeline, abundance information of each of the member taxon in the community will be inevitably lost. Although some correlation of NGS reads and taxonomic abundance has been shown in mixed nematode samples [36] and diet analyses [37,38], such success will largely depend on the phylogenetic diversity of taxa in question and the performance of primers applied, which is challenging for most animal groups used as biological proxies (e.g., macroinvertebrates). Protocols such as DNA capture and environmental DNA shotgun sequencing have been proposed by Taberlet et al. [39] to bypass PCR. Some preliminary work also showed the feasibility of applying this approach on eukaryotic diversity [40]. But systematic metabarcoding studies on real eukaryotic communities that are independent of PCR are lacking.

In this study, we aim to develop a new NGS pipeline that is independent of PCR amplifications (Figure 1), while still enabling molecular identification of insects at the species level, using bulk insect samples for the proof of concept. Two major challenges for the complete elimination of PCR amplification need to be resolved first: (a) detection of target DNA sequences at low quantity and (b) taxonomic assignment based on short NGS reads. A 650 bp sequence fragment on the 5^′^ end of the *mtCOI* gene has been widely adopted as the DNA barcode region for identifying animal species since its initial proposal [13]. Although mitochondria are found in vast copy numbers in metazoan animals, mitochondria (MT) nucleotides only account for a small fraction of the total DNA compared to nuclear sequences (e.g., 0.05% in *Bombyx mori*[41]) and sequences of microbial origin in the DNA soup. The ultra-deep sequencing capacity of the Illumina sequencing platform provides an opportunity to examine the feasibility of detecting minute trace of mitochondrial sequences directly from genomic DNA mixtures. For example, each sequencing run of the HiSeq 2000 sequencer is able to produce approximately 600 Gbp, equivalent to 200 human genomes, which is more than 1,000X as much as the capacity of the Roche 454 GS FLX + platform (see review [20]). On the other hand, the current Illumina sequence reads for HiSeq 2000 can only reach up to 150 bp. This short sequence length presents a limitation to the full utilization of full-length DNA barcodes (approximately 650 bp for animal *COI* barcodes) available globally (e.g., through the International Barcode of Life initiative [42]). Although a very short piece of the standard barcodes (“mini-barcode”) of only 130 bp demonstrated reliable taxonomic resolution for a number of animal groups [43], longer sequences are always preferred for improved identification power. Therefore, we need informatics solutions to assign species-level identity based on mixed short Illumina reads.

To overcome these two major hurdles, the new pipeline employed in the current study involves pre-sequencing enrichment of mitochondria followed by total DNA extraction, shotgun sequencing of isolated total DNA using the Illumina HiSeq 2000, and taxonomic identification of NGS reads. Two different approaches can be applied to assign species-level identity: (1) if an *a priori* barcode reference library exists for the investigated fauna, NGS reads are mapped to the reference sequences following defined criteria; and (2) when this reference library is absent, *de novo* assembly of NGS reads into mitochondrial gene fragments, especially the *COI* barcode region, is employed, followed by gene annotation, to ensure accurate detection of taxa from the mixed bulk sample. Based on the promising results from our *in silico* simulations using 209 insect mitochondrial genomes obtained from GenBank (details of the design and results of the simulation are provided in Additional file 1: Appendix S1), we apply the new Illumina pipeline to analyze real bulk insect samples. A preliminary study was first performed to gain a basic understanding for the required scale of Illumina sequencing. Details were summarized in Additional file 2: Appendix S2. A formal sample was subsequently sequenced and analyzed with ultra-high sequencing volume (15.5 Gb) to systematically test and discuss this NGS pipeline on biodiversity study in the following text.

The main question of the present work is: can PCR amplifications be avoided in analyzing arthropod biodiversity using the Illumina HiSeq 2000 platform? Specifically, we focus on: (1) how much Illumina sequencing is required with regards to a reliable estimation for species composition from bulk insect samples at a given species setting and (2) what informatics tools can facilitate such analysis? To answer these questions, we use an empirical approach to evaluate the accuracy of species composition estimates (quantifying true positives, false negatives, and false positives). We also measure the impact of two analysis strategies (i.e., the presence or absence of a reference barcode library) for the focal fauna on taxonomic discovery. Additionally, exploiting another potential benefit of abandoning PCR amplifications, we explore the indication for relative abundance of each taxon present in the bulk sample. Lastly, we discuss the pros and cons of this new pipeline and provide suggestions for potential improvements to achieve its real-world application.

## Data description

Seventy-three insect individuals were collected from a mountainous habitat in the sub-tropical region of China (22°36^′^01.38”N, 114°16^′^00.76”E, approximately 340 m above sea level [ASL]) on October 5^th^ 2011 and preserved in 99.5% ethanol at 4°C for one month. Every individual was identified morphologically by authors of the paper to the finest taxonomic level as much as possible. Genomic DNA was first extracted from a single leg of each specimen for DNA barcoding. Standard *COI* barcodes for all specimens were individually Sanger sequenced and compared against the Barcode of Life Data Systems for taxonomic confirmation. Subsequently, after mitochondrial enrichment using differential centrifugation, total genomic DNA was extracted from homogenized tissues for mixed insect sample. The DNA library of an insert size of 200 bp was constructed for the bulk sample and then sequenced on an Illumina HiSeq 2000 analyzer at BGI (Shenzhen, China) using 100 bp paired-end (PE) sequencing, following the manufacturer’s instruction. The DNA library was analyzed using an ultra-deep sequencing strategy (approximately 15.5 Gb) to examine the impacts of sequencing volume on biodiversity recovery.

Sequences containing adaptor contaminations (with > 15 bp matched to the adapter sequence) and poly-Ns were filtered out. PE reads were removed from subsequent analyses if >10 bases were of low quality scores (<20, i.e., sequencing error rate > 1%). A new dataset containing only high-quality reads was created after these filtering steps, and is available from the *GigaScience* Database [44].

## Analyses

### Reference barcode library

A total of 37 Operational Taxonomic Units (OTUs) were reported based on morphological identification and DNA barcoding (Sanger sequencing of the standard *COI* barcode region) of the 73 insect specimens. The basic information including taxonomic composition (in the form of molecular OTUs [MOTUs]) and data size, has been summarized in Table 1. All MOTUs are shown in a neighbor joining (NJ) tree in Additional file 3: Figure S1. A total of 69 standardized *COI* barcodes were successfully sequenced and taxonomically assigned. Four individuals, representing 3 morphological species (identified morphologically as 2 dipterans, and 1 hemipteran, respectively), failed in multiple rounds of PCR amplifications. But additional individuals for all 3 morpho-species had been successfully sequenced and were already represented in the reference barcode library (OTU41, OTU48, and OTU58). Since reference barcodes were not available for most of China’s insect fauna, most specimens were only identified to the family or order level based on morphological characters (Additional file 4: Table S1). An arbitrary threshold of 2% was subsequently applied to delineate MOTUs, yielding 37 MOTUs (Additional file 4: Table S2).

**Table 1:** **Sample composition, sequencing information and *COI *recovery rates of the bulk insect sample**

Number of Individuals	73
Number of *COI* barcodes obtained	69
Number of MOTUs (2%)	37
Raw data size (Gb)	15.5
High quality data size (Gb)	13.2
Discovery rate (with reference)	97%
Discovery rate (no reference)	92%
Assembly coverage rate (% MT genomes)	74%
Total length and percentage of *COI* genes ^1^	51,768 (96%)
Number of assembled mitochondrial genes ^2^	613

^1^ The total length (bp) of assembled *COI* genes and the percentage of assembled *COI* sequences for 37 full-length *COI* genes (~ 1,530 bp for each species).

^2^ Note that a small portion of the genes were assembled into two scaffolds.

### Taxonomic recoveries

We developed two independent bioinformatics pipelines for when the *COI* barcode reference library is available for the focal fauna and when one is not available.

#### Reference-based method

Sequence coverage (percentage of a reference sequence matched by Illumina short reads) was used as a criterion for taxonomic detection (Additional file 1: Appendix S1, Additional file 3: Figure S2). Sequence coverage was calculated by aligning full length PE reads to the reference. A reference sequence was considered matched only when > 90% nucleotides were covered by query reads at ≥ 99% identity (Additional file 3: Figure S2).

By aligning high-quality reads to the Sanger library, we detected 36 MOTUs out of 37 references (97.3%). The only exception was one species (OTU51) that was represented by a single specimen with a body length < 2 mm (Additional file 4: Table S2, Additional file 3: Figure S3).

#### Reference independent method

We also tested the scenario where barcode reference libraries were unavailable *a priori* for the investigated fauna, an assumption that is still true for most regions of the world. High-quality Illumina reads were *de novo* assembled into scaffolds using SOAPdenovo2 [45,46], which were then annotated for mitochondrial genes using methods provided in the Methods section. Through ultra-deep sequencing of genomic DNA and *de novo* assembly of all shotgun reads, a total of 370 mitochondrial scaffolds were successfully assembled with an N50 of 1,721 bp and a maximum length of 15,326 bp (Table 2). A total of 42 scaffolds contained homologs of the standard *COI* barcode region, 4 of which were screened out as bacterial sequences. Two of them had *Wolbachia* origin, the well-known microbial symbionts frequently found in many insects, while the other 2 belonged to *COI* from Legionellaceae and *Cytochrome O Ubiquinol Oxidase Subunit 1* from Bartonellaceae, respectively. The remaining 38 scaffolds were identified as insect *COI*. However, 3 relatively short scaffolds were aligned to the 5^′^ end of the barcode region, while another 7 were aligned to the 3’ end, without overlapping in between. Given that the reference barcodes were not used in this pipeline, we only chose fragments closer to the 3^′^ end for the consequent analyses, bringing the total detected MOTU number to 35. Such a conservative strategy might underestimate the real species richness if the fragmented scaffolds represented different species, but would reduce the changes of “biodiversity inflation”. To evaluate the assembly completeness of this pipeline, these 10 fragments were compared to the Sanger reference barcodes. We concluded that the 3 fragments aligned to the 5^′^ end belonged to 3 MOTUs that were already represented by corresponding 3′ fragments. Therefore, no species with a sequence had been dropped out in our analysis. One novel MOTU (Novel1, absent from the reference barcode library) was found in our NGS result. The source of this taxon (Lepidoptera) is unclear. Judging from its low coverage (5.6X), the source DNA must have been in low quantity, presumably from residual tissues left in the bulk sample (e.g., eggs, scales, food item). In summary, among the 37 OTUs presented in the bulk sample, 34 (91.9%) were recovered by our reference independent approach, while 1 novel OTU was found (Additional file 4: Table S2, Additional file 3: Figure S3).

**Table 2:** **Results of *de novo *assembly for the 35 detected *COI *scaffolds and 370 mitochondrial scaffolds**

	**Length of *COI *scaffolds (bp)**	**Length of Mitochondrial scaffolds (bp)**
Minimum	403	106
Average	3,544	1,065
N50	5,674	1,721
N90	1,890	444
Maximum	15,326	15,326

**Figure 2:**
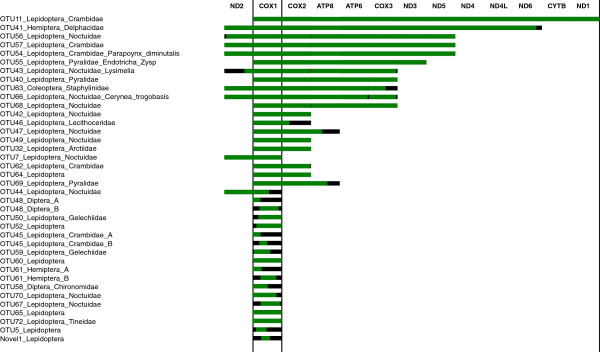
**Assembly results of mitochondrial genes.** Green bars represent successfully annotated and aligned genes and black bars represent gaps. Additional genes (non-*COI*) are also assembled and annotated (Table 1), but cannot be aligned to same scaffolds containing *COI*. These are not shown in the figure. The suffix (A / B) indicates the corresponding reference was assembled into different scaffolds.

The assembled *COI* barcode sequences are of high quality. The average length of the final 35 scaffolds containing the insect *COI* barcode region was 3,544 bp (Table 2), while 33 of them exceeded 500 bp. About half of these scaffolds expanded beyond the *COI* gene. Of the 34 scaffolds found in the input reference, 23 had 100% match to Sanger references, while another 10 were aligned to references at identity ≥ 99%. In most cases, these minor mismatches (1–2 bases per 658 bp) had no effect on taxonomic assignment. This result indicated that homologous mitochondrial sequences in the bulk sample barely impeded assembly at the given species set. Only 1 scaffold showed relatively lower (96%) identity to the corresponding reference barcode. Given its relatively low sequencing depth (4.7X), these mismatches were likely caused by assembly errors. Moreover, we successfully assembled many non-*COI* genes (Table 1). Gene assembly and annotation results are illustrated in Figure 2.

### Impacts of sequencing volume on discoveries of taxonomic richness

**Figure 3:**
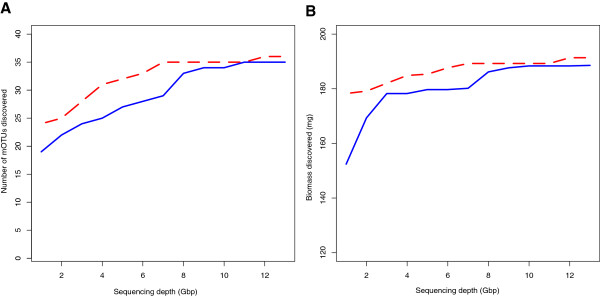
**Rarefaction curves for discoveries of (A) MOTUs and (B) biomass.** With increasing sequence quantity, discovery rates for MOTUs and biomass are both improved, using a reference-based method (red dashed line) and a reference independent method (blue solid line).

Through ultra-deep sequencing, 97% and 92% of the taxa from the bulk insect sample were recovered by reference-based and reference-independent methods, respectively (Table 1, Additional file 4: Table S2). To evaluate the influence of sequencing volume on the success of taxonomic recovery, we plotted the number of discovered OTUs and biomass against sequence data at a 1 Gb interval, for both reference-based and reference independent methods (Figure 3A and 3B). Apparent improvements on species discovery rate were observed in both methods when sequencing volume was increased. The reference-based method exhibited better taxon discovery when sequencing volume was less than 8 Gb, proving the value of barcode reference libraries in NGS biodiversity analyses. However, if the sequencing volume was large enough (e.g., 8 Gb in the current diversity composition and lab protocol setting), sequencing for nearly all mitochondrial DNA fragments in the mixed sample had reached saturation. Although new taxa were still detected with more sequencing, both curves seemed to have reached a plateau at approximately 8 Gb, where 95% and 89% of the total taxa had been already revealed by the reference-based and reference-independent methods, respectively (Figure 3A). Only a few new taxa with very small biomass were further picked up by additional sequencing (from 8 to 13 Gb).

Between the reference-based and reference independent methods (Figure 3A), it was also obvious that the increase of sequencing volume made larger improvement on the latter, where sequence assembly was a critical step in the bioinformatics pipeline. Our experience showed that an average sequencing depth of 10X would generally produce a good assembly. In fact, the number of MOTUs with sequencing depth < 10X was decreased from 24 to 9 at 1 Gb and 13 Gb, respectively.

### Correlation between sequencing volume and biomass

**Figure 4:**
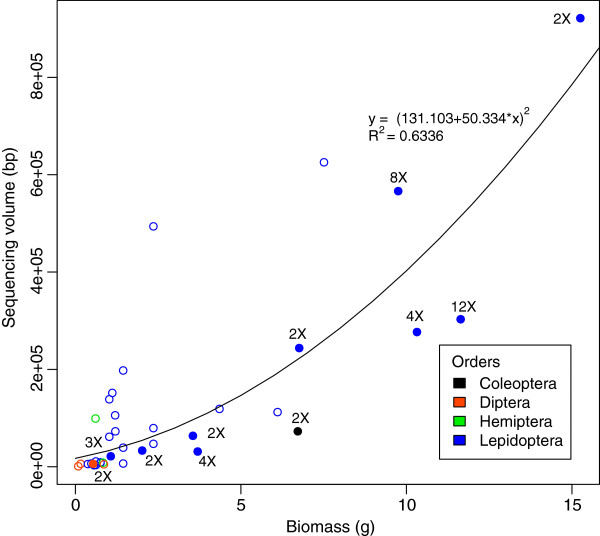
**Correlation between biomass and data volume.** The sequence volume for each given taxon was measured by number of base pairs. The corresponding biomass was estimated based on the body-length. Hollow circles represents the taxa with a single individual, while solid circles represents the taxa with multiple individuals. Number of individuals in the corresponding MOTU is also labeled on the solid circle. Small taxa were typically sequenced at lower sequencing volumes. But those represented by multiple individuals had higher sequencing volume. The increase of sensitivity for these small insects was likely a consequence of the increased total biomass for the given species.

The removal of PCR amplifications from the NGS pipeline creates an opportunity to reveal abundance information for taxa present in the bulk sample. In principle, the sequence volume of a given specimen should be correlated to the total copy number of mitochondria belonging to that individual, assuming that everything else (tissue blending, DNA extraction, etc.) is not biased to favor any particular taxonomic groups. Several factors may determine the total quantity of mitochondria, including heterogeneity in mitochondrial copy numbers in different cell types, total cell numbers, body size, biomass, some of which could be correlated to each other. Among these, biomass seems to be a good surrogate and can be calculated based on body-length, which is much easier to measure in practice. Therefore, we evaluated the correlation of sequencing volume and biomass for taxa in the bulk insect sample. There seems to be an obvious correlation between sequencing volume and total biomass for a given species (Figure 4), which is not affected by the choice of equation used for estimating biomass from body-length (Additional file 3: Figure S4). This pattern has also been confirmed in all insect orders we tested. If multiple individuals are present, this obviously increases the total biomass of that species present in the sample. Indeed, we found that species with multiple individuals in the sample had more sequence coverage than those of similar size, but with a single individual (Figure 4, solid vs. hollow dots). Such results are highly congruent with the observation that it is generally more difficult to detect smaller specimens at any given sequencing volume (Additional file 3: Figure S5).

## Discussion

### Ultra-deep sequencing enables detection for small trace of mitochondrial sequences

NGS technologies have been employed in biodiversity analyses for varied environments, with proposed advantages in throughput and cost. However, most of these studies have been based on PCR amplifications, especially in eukaryotes, which typically possess large-sized genomes. Although the incorporation of PCR amplifications has the obvious advantage in producing sufficient amplicons of the targeted gene fragments, such practice often introduces taxonomic biases due to varying primer efficiencies across taxa and sequence errors caused by mismatches of complementary strains to the DNA templates [7,21,23,26,28,47]. The main challenge in reducing these biases by eliminating PCR is to ensure that the community diversity can be accurately measured through the small amount of mitochondrial sequences in the mixed DNA soup. In this study, this objective is achieved through mitochondrial enrichment and deep sequencing.

The mitochondrial enrichment protocol employed in this study clearly has room for improvement. The whole enrichment procedure yielded mitochondrial DNA accounting for only 0.53% of the total raw data (15.5 Gb), which was approximately 10X higher than the original judging by the corresponding MT sequence proportion found in the silk worm (0.05% [41]). A much smaller percentage (0.03%) belonged to *COI* genes. However, even with this small fraction of the total sequence data, 74% of the total mitochondrial genomes presented in the bulk sample had been successfully assembled, while 96% of the total *COI* sequences had been covered (Table 1) – a clear benefit of the deep sequencing capacity of Illumina’s HiSeq 2000 sequencer. This minute proportion of mitochondrial sequences contains almost 5 million base pairs of high-quality data belonging to *COI* genes, equivalent to >7,000 full-length DNA barcodes, which is almost comparable to the total raw data capacity of an entire 454 run. On average, each of the 37 member species in the insect community has been covered by more than 200 full-length barcodes equivalence of sequence reads. It is this high sequence coverage that has ensured species recovery at high accuracy.

### PCR-independent method delivers species recovery at high fidelity

These *COI* sequences detected by our NGS pipeline enabled recoveries for 97% (with a reference barcode library) and 92% (without reference) of the total taxa in the bulk insect sample. To our knowledge, these results represent the highest rates of “true positive” discoveries in all published NGS analyses for arthropod diversity that has a controlled reference.

All taxa missed by the NGS pipeline (false negatives) are characterized by low biomass and are subsequently covered by only low sequencing depth. Only 1 and 3 taxa were not detected by the reference-based and reference-independent methods, respectively. All missed specimens had a body length < 5 mm (Additional file 3: Figure S5).

Our pipeline, which eliminates PCR amplification, rarely picks up novel taxa, and thus avoids the “taxon inflation” common to other NGS-based biodiversity assessments [16,33]. Only 1 detected taxon identified molecularly as Lepidoptera was found absent in the reference barcode library. Based on its good assembly quality (yet low read coverage) and highly congruent amino acid sequence composition compared to lepidopteran barcodes in BOLD, we argue that this novel sequence is not a real ‘false positive’. The exact source for this ‘novel taxon’ is unclear, but possibilities might include gut content, undetected tissue (damaged pieces, eggs, small body-size, etc.) in the insect mixture, environmental DNA and so on. The extremely low rate of novel taxa is due to the stringent algorithm involved in the matching criteria based on sequence coverage (reference-based) and *de novo* assembly of Illumina shotgun reads (reference independent), which eliminates nearly all sequence errors.

### A potential way to obtain relative abundance?

The correlation between sequencing volume (the number of nucleotides) and biomass of a given species has not only produced biological production information of the insect community, but also provided a possible solution to investigating relative abundance of each arthropod taxon present in the bulk sample. This possibility relies on the availability of a reference barcode library for the focal fauna and a database for the range of biomass of each of the arthropod species in this fauna. Presumably, the number of individuals of a given species can be estimated by the total biomass – that was calculated from the total nucleotide base pairs of this species – divided by an average biomass of the target species. Apparently, a well-designed test, including a wider range of taxa with varied biomasses, is needed to draw solid conclusion on the feasibility of this approach, which is beyond the scope of this paper. Nevertheless, our new NGS protocol, especially the elimination of PCR amplifications, has created an alternative way to quantify abundance (although as a statistical estimation) – the critical information concerned in ecological studies.

### Further improvements for applications in real-world scenario

Admittedly, the insect bulk sample tested in the present study only represents biodiversity examples at a moderate level. Although our diversity scale is comparable to some recent work (e.g., [33,48]), community samples consisting of more complex components (e.g., more than hundreds of species from wider taxonomic ranges) can be expected in many real-world sampling efforts. Nonetheless, our work provides an invaluable first-hand knowledge on the requirement of sequencing volume when PCR is avoided. Based on our findings in the correlations between sequencing volume and discoveries of MOTU and biomass (Figure 3), a simple extrapolation suggests that it may require multiple lanes of Illumina HiSeq data to handle bulk insect samples containing hundreds of species. Therefore, a more efficient mitochondrial enrichment procedure is desired to enlarge the sampling capacity per Illumina lane. Provided that the current sequencing cost for Hiseq 2000 (based on the list price from the manufacturer) is approximately $40 per gigabase, the sequence yield per run is 600 Gb [49], and the sequencing volume needed to reveal insect richness from bulk samples (based on empirical data of this paper), we calculate the average cost for discovering a single insect species is less than $20. This cost is already very close to that of classic DNA barcoding of an individual specimen using Sanger sequencing. Given the trajectory of NGS cost reduction over the past years, it is reasonable to expect wide adoption of a PCR-free Illumina shotgun approach in routine biodiversity studies.

Furthermore, alternative protocols for DNA preservation and mitochondrial isolation are expected to greatly increase the portion of mtDNA sequences in the total DNA sample, which can be translated into less sequencing and lower cost. Preservation media, such as DESS [50], that help to maintain the integrity of mitochondria and circular DNA, coupled with enzymes digesting linear DNA may help to vastly remove nuclear and even bacterial DNA. A DNA-capture-based method might perform better for small volume of targeted DNA [51]. While our mitochondrion enrichment protocol is able to handle specimens preserved in ethanol for 1 month, a DNA-capture-based method might be more appropriate for older specimens with more highly degraded DNA. For bulk samples containing arthropods with a large range of body sizes, a pre-sorting of large samples from smaller ones followed by sub-sampling and re-mixing (tissue normalizing) should help to improve the detection of small individuals, given the total sequencing volume remains at the same level.

It is also clear that a comprehensive DNA barcode library and a genomic database in general will significantly improve our analytical efficiency in the NGS analysis for biodiversity composition. These databases will not only improve species detection rates (reference based vs. reference independent methods) for arthropods, but also help to reach a better understanding of the presence of the much broader diversity (e.g., algae, bacteria, virus) in the bulk sample.

In summary, the ultra-high sequencing capacity of the Illumina HiSeq 2000 platform provides a new NGS solution that avoids the use of PCR amplifications of particular gene markers in arthropod biodiversity analysis. This new pipeline not only reveals species richness in high fidelity, but also creates a possibility to reveal relative abundance of each taxon present in the bulk sample. The ability to detect small arthropod specimens and its tendency to avoid most, if not all, false positives has suggested its great potential in biodiversity related surveys, such as in biomonitoring programs. However, it is crucial to improve mitochondrial enrichment to ensure applications of the new NGS pipeline in analyzing more complex biodiversity settings.

## Methods

### Sample collection

In this study, we chose insects as the model system to test our new pipeline because they had been widely used in environmental quality assessments and biomonitoring programs [52]. Insect samples were collected using a 15 W black-light trap (Bioquip, CA, USA) and a white pan filled with 99.5% ethanol. A mountainous habitat, Beishan, close to the BGI’s headquarters in Shenzhen, China, was sampled on October 2 (Preliminary Sample, 22°35^′^38.94”N, 114°15^′^54.64”E, approximately 55 m ASL) and October 5 (Formal Sample, 22°36^′^01.38”N, 114°16^′^00.76”E, approximately 340 m ASL) in 2011. The two collecting sites differed around 290 m in elevation with similar vegetation. A total of 89 and 73 individuals were collected, respectively, and preserved as two separate bulk samples at 4°C in the laboratory. All specimens were individually barcoded, photographed and measured for body-length. Taxonomic identification was achieved using morphological characters and by comparisons of *COI* barcodes against the Barcode of Life Data Systems [53].

### Mitochondrial DNA enrichment and extraction

Mitochondrial enrichment was conducted using differential centrifugation [54] with protocols optimized for mixed insects. Bulk samples were preserved in 99.5% ethanol at 4°C for 1 week (Preliminary Sample) and 1 month (Formal Sample) before isolation. All insects were removed from ethanol and washed with chilled 1X MS buffer (210 mM mannitol, 70 mM sucrose, 5 mM TrisHCl, 1 mM EDTA) to remove residual ethanol. Large insects were firstly sliced into small pieces to facilitate the homogenization process. All individuals were then pooled and homogenized using an IKA T-10 basic homogenizer (IKA, Germany). To alleviate mitochondrial deterioration, homogenation was performed in chilled 1X MS buffer (10 times larger than the volume of the total tissue sample) in a 50 mL polypropylene conical tube for 5 minutes. The homogenate was then centrifuged at 1,300 g at 4°C to remove nucleic and cellular debris. Collected supernatant was centrifuged at 17,000 g for 30 min to enrich mitochondria.

The isolated mitochondria were lysed in the mitochondrion lysis buffer (0.15 M NaCl, 10 mM TrisHCl, 1 mM EDTA), with 5% SDS and 0.5 mg/ml proteinase K. The extracted total DNA was purified using phenol-chloroform and isoamyl alcohol mixture. DNA was isolated using NH_4_Ac and absolute alcohol. Finally, DNA was dissolved in TE buffer (10 mM Tris HCl, 1 mM EDTA).

### Construction of DNA barcode references

Two legs of each specimen were removed for genomic DNA extraction and subsequent acquisition of *COI* barcodes, with the rest of body tissues saved for NGS analyses. A DNA release method with Cell Lysis Solution and Proteinase K was used to release genomic DNA [55], following standard barcoding protocols used by the Canadian Center for DNA Barcoding [56]. Two different primer sets were used in a two-tiered PCR amplification: LepF1/LepR1 [57], followed by LCO1490/HCO2198 [30] when the first round failed. Individual DNA was amplified with a 25 μl-volume reaction, containing 1 μl of genomic DNA, 1 μl of each forward and reverse primers, 16.2 μl of H_2_O, 2.5 μl of 10X buffer, 3 μl of dNTP mix, and 0.3 μl of Ex-taq DNA polymerase (TaKaRa Biosystems). PCR products were visualized using 2% agarose gels. Positive PCR products were then Sanger sequenced using an ABI 3730XL DNA sequencer at BGI.

A NJ tree was built for all Sanger sequences using MEGA V5.0 [58] and Interactive Tree Of Life [59] with a Kimura-2-Parameter setting and pair-wise distances. This *COI* tree based on sequence similarity shows sequence divergences within and among MOTUs and roughly represents taxonomic diversity.

### Coverage calculation of reference based method

*COI* sequences obtained in this study via individual Sanger sequencing were used as the reference for taxonomic assignments of the short Illumina reads. Sanger references were first clustered into MOTUs using an arbitrary threshold of 2%. Shotgun reads were then aligned to the Sanger references using the program BLASTN [60], allowing 99% identity and full length alignment of the PE reads. The qualified reads was then used to calculate the overall coverage of reference barcodes using SOAP coverage [61]. A reference sequence was considered matched only when > 90% coverage was reached.

### Assembly and annotation of reference independent method

High quality Illumina reads (including non-*COI* mitochondrial reads) were assembled into contigs and scaffolds using the genome assembler program SOAPdenovo2 [45,46], independent of a *COI* library. Through the construction of de Bruijn graphs, contigs were built using a k-mer with a size of 61 bp and resolving repeats by reads. Reads were then aligned to contigs with a 45 bp k-mer and unmasking contigs with high coverage. The paired-end information embedded in sequence reads was also referred in scaffold constructions.

All mitochondrial protein-coding genes, including the *COI* barcode region, were annotated using homolog prediction. Assembled scaffolds were aligned to a set of 244 complete mitochondrial genomes obtained from GenBank using TBLASTN with an e-value ≤10^−5^. The BLAST results were then used to determine gene ontology (e.g., mRNA and coding sequence regions) using Genewise [62], which was confirmed by DOGMA [63]. Ribosomal RNA genes were annotated using BLAST by comparing to the 244 mitochondrial genomic rRNA database with an e-value ≤10^−5^ and annotated when its length was ≥ 200 bp or ≥ 300 bp, for the 12S or 16S rRNAs, respectively. Genes that could not be annotated through these steps were then manually annotated by aligning to corresponding gene sequences of the 244 mitochondrial genomes and by examining amino acid translation. For example, the *ATP8* gene is relatively short (typically ~159 bp) and is difficult for automated annotation. In addition to the *ATP8* genes obtained using the homolog prediction method, we further aligned regions between annotated *COX2* and *ATP6* and manually identified more *ATP8* genes. The highly conservative protein sequences translated from all annotated *ATP8* genes confirmed both assembly and annotation.

The accuracy of assembly was validated by PCR and Sanger sequencing for a set of randomly selected genes assembled and annotated from the preliminary sample (Additional file 2: Appendix S2, Additional file 3: Figure S6). All Sanger results were identical to the assembly. PCR validation was only conducted on preliminary sample because the formal sample was expected to contain assemblies of even higher quality due to its much larger volume of data.

### Biomass estimation and the correlation analysis

Different biomass equations [64-66] were utilized to calculate biomass based on body size. No significant differences were observed among these methods based on R^2^ and P value of coefficients (Additional file 3: Figure S4). In the main text, we presented biomass estimation using one of these equations: ln(Y) = ln(a) + b*ln(X), where X represented length*width and Y referred to biomass; a, b were parameters for different insect orders [64]. Body sizes were measured using a metric scale set beside the insect specimen during photographing. Biomass was categorized into different orders. We plotted the sequence volume (in base pairs) of a given species to the total biomass of that species (including all individuals of the same species). To examine the correlation between sequencing volume and biomass, correlation analysis was performed on the datasets. Results were tested using F statistic of regression equation and t statistic of regression coefficient test. The *P* value of both statistics were < 0.001, reaching a significant level.

#### Availability and requirements

This pipeline has been adapted from SOAPdenovo2 [46] and therefore distributed under the same license terms.

• **Project name**: zhou2013 (PCR-free pipeline for DNA metabarcoding)

• **Project home page**: https://github.com/gigascience/papers/tree/master/zhou2013

• **Operating system**(**s**): Unix, Linux, Mac

• **Programming language**: PERL

• **Other requirements**: GCC version ≥ 4.4.5

• **License**: GNU General Public License version 3.0 (GPLv3)

• **Any restrictions to use by non**-**academics**: none

## Availability of supporting data

The raw sequence data in fastq format, assembly and annotation results are available from *GigaScience* Database [44] and the short read archive SRA067357. In order to facilitate readers to repeat the experiments the annotation pipeline is available from the *GigaScience* Database [67].

## Abbreviations

ASL: Above sea level; bp: Base pair; COI: *Cytochrome c oxidase subunit 1*; mt: Mitochondria; MOTU: Molecular operational taxonomic unit; NGS: Next generation sequencing; NJ: Neighbor joining; OTU: Operational taxonomic units; PE: Paired end; PCR: Polymerase chain reaction.

## Competing interests

The authors declare that they have no competing interests.

## Authors’ contributions

XZ YL SL conceived and designed the experiments. QY XS RF JL YL MT performed the experiments. XZ YL LZ QH analyzed the data. XZ YL SL contributed reagents/materials/analysis tools. XZ YL wrote the paper. XZ YL SL edited the manuscript. All authors read and approve the final manuscript.

## Supplementary Material

Additional file 1 **Appendix S1. ***In silico* simulation of taxonomic detection via Illumina shotgun reads using reference-based and reference independent methods. Click here for file

Additional file 2 **Appendix S2.** Analyses of taxonomic recovery for preliminary sample. Click here for file

Additional file 3: Figure S1 Taxonomic composition of bulk arthropod Preliminary samples & Formal sample. A Neighbor-Joining tree of *COI* barcode sequences extracted from individual specimens. The NJ tree was constructed using MEGA 5.0 using a distance method and defaultparameters. Tree terminals were collapsed into triangles using tools provided by the Interactive Tree of Life using an arbitrary threshold of 2%, as a rough estimation for the species diversity. MOTUs found in preliminary study and the formal sample are marked in red and blue colors, respectively. Four species found in both samples were marked in green color. **Figure S2.** The schematic demonstration of the matching criteria employed in the reference-based method. Only when the coverage of a reference by Illumina reads is evenly distributed, as shown in Reference 1, this taxon is considered successfully detected. Specifically, >90% of the reference sequence has to be matched at >99% similarity, where the reference coverage rate. **Figure S3.** Venn Diagram for the MOTU discovery for using reference-based and reference independent methods. **Figure S4.** Correlation between biomass and data volume with different biomass equations. Two more biomass equations were tested. (A): biomass was calculated based on the method of Ganihar et al. [65] (B): biomass was estimated with the parameters of Hódar et al. [66] All the P values of coefficients are significant (<0.001). **Figure S5.** Ranges of arthropod body-sizes that were detected and missed at given sequencing volumes. Apparent divergences of body-sizes between detected and missed taxa were observed along the increase of sequencing volume in both methods. Deeper sequencing enabled detections of smaller taxa. **Figure S6.** Assembly results for all *COI* genes and long scaffolds containing additional mitochondrial genes in Preliminary sample (2.5 Gb). Dark green bars represent successfully annotated genes; black bars represent gaps; and light green bars represent genes confirmed by PCR validation. Non-*COI* genes that cannot be assembled into same scaffolds containing *COI* are not shown in the figure. **Figure S7.** Thresholds of taxonomic resolution using simulated Illumina shotgun reads. The distance tree is built based on 24 species from 6 insect genera, each of which are represented by multiple closely related species. The red line indicates the taxonomic resolution using the proposed NGS methods at 100X sequencing depth. Gray line indicates the species identify resolution may decrease to about 1%, with the increase of sequencing depth. Click here for file

Additional file 4: Table S1 MOTUs found in preliminary sample and formal sample and the relevant taxonomic identification based on morphology and DNA barcodes. **Table S2.** Taxonomic recovery for using reference-based and reference independent approaches. Click here for file
