# Supplementary material for: Ultra-deep sequencing enables high-fidelity recovery of biodiversity for bulk arthropod samples without PCR amplification
Source: Gigascience. 2013 Mar 27;2:4. doi: 10.1186/2047-217X-2-4 (PMC3637469; doi:10.1186/2047-217X-2-4)
Supplement: Additional file 1 — Appendix S1. In silico simulation of taxonomic detection via Illumina shotgun reads using reference-based and reference independent methods. [file 2047-217X-2-4-S1.docx]

**Appendix S1. *In silico* simulation of taxonomic detection via Illumina shotgun reads using reference-based and reference independent methods**

The Illumina HiSeq 2000 platform produces 100 bp short reads when using the 100 paired-end (PE) sequencing strategy. The major challenge for species identification from a pooled sample using NGS technology is to distinguish shotgun reads from each other and to assign them to the corresponding reference barcodes when possible. The success of this practice is dependent on the taxonomic resolution that can be delivered by these short DNA fragments as well as the attribution of the insect community in question. Although a 130 bp fragment of the full-length DNA barcode – “mini-barcode” – provides promising resolution at the species level for a number of insect groups [43], longer DNA strains are always desired. On the other hand, the phylogenetic relatedness of the co-occurring taxa and their relative abundance may both pose impacts on the efficiency of the taxonomic identification using short sequence reads. In addition, different sequencing depths (total sequence base pairs divided by total length of targeted genes) may alter the taxonomic resolution for a given set of bulk sample.

**Material and Methods**

We propose solutions for two different scenarios (i.e., where a reference *COI* barcode library is available for the investigated fauna, and where it is not). The protocols and criteria of species identification employed by both methods have been explained in the Methods session in the main text. Briefly, when the reference library is present, Illumina shotgun reads can be aligned to the reference sequences at > 99% identity for the full PE reads. Only when > 90% length of a reference is matched by shotgun reads, this reference is considered detected in the bulk sample. Alternatively, if the reference library is unavailable, Illumina reads can be *de novo* assembled into contigs and scaffolds using SOAPdenovo [45,46]. *COI* barcode sequences are identified from these longer DNA fragments based on annotation and amino acid sequences. In real community samples, non-arthropod barcodes (microbes and other contaminates) can be eliminated by searching against the Barcode of Life Data Systems (BOLD) and GenBank. The final set of *COI* MOTUs can be further assigned to the lowest taxonomic identity through BOLD or used directly as a proxy of the species-level richness.

To test the feasibility of these methods, we created six mock-up samples, each with species of varied relatedness and abundances, from 209 complete insect mitochondrial genomes obtained from the GenBank (Table S3). Although these taxa cannot represent real insect diversity in natural communities, this dataset contains a good variety of insect lineages, including a few genera with closely related species, thus provides a good opportunity to examine the proposed methods.

1. **Reference based method**

*Reference barcode library*

In the simulation process, standardized *COI* barcode regions obtained from full mitochondrial genomes were used as reference sequences. These barcode sequences were identified by searching for conservative primer regions flanking the barcode region, using the Folmer primers (LCO1490 and HCO2198, Table S4) [30].

*“Shotgun” reads*

Mitochondrial genomes in each mock-up mixture were randomly broken into 180-bp fragments and were “sequenced” using a 100 bp PE sequencing strategy. One percent sequencing error rate was assigned to better simulate the real sequencing process [68].

*Aligning method and matching criteria*The program BLASTN was employed [60] to align short reads to reference barcode sequences (standardized *COI* barcodes obtained from whole mitochondrial genomes), allowing 99% identity and full length alignment of the PE reads. SOAP coverage [61] was then used to calculate the overall coverage of reference barcodes (Figure S2 in additional file 3). A reference sequence was considered matched only when > 90% coverage was reached.

*Mock-up samples*

For the reference-based method, we created 3 sets of *in silico* simulations (Table S5): (1A) all 209 species were pooled at even abundance, which was sequenced at 100X sequencing depth to gain basic understanding of the performance of the proposed informatics protocol; (1B) 5 species that each had multiple closely related species presented in the reference were pooled at even abundances and sequenced at 100X; and (1C) the same species set from (1B) were sequenced at 1,000X depth to explore the influence of sequencing depth on taxonomic resolution. The full set of reference barcodes consisting of 209 species was made available for species identification for all 3 simulations.

1. **Reference independent method**

*De novo assembly and annotation*

In the reference independent method (Table S5), shotgun reads created in the same way described above were *de novo* assembled into longer DNA sequences (contigs and scaffolds), using the program SOAPdenovo [45,46]. The barcode regions were then identified using the annotated amino acid sequences.

*Mock-up samples*

Three mock-up samples were created for the reference independent method: (2A) 15 mitochondrial genomes were pooled at varied abundances and were sequenced at 100X to test the efficiency of the assembly approach; (2B) 4 closely related *Drosophila* species were pooled at even abundances and were sequenced at 10X to understand the taxonomic resolution when a reference barcode library was absent; (2C) the same set of 4 *Drosophila* species at varied abundance.

**Results**

1. **Reference based method**

In simulation 1A, all 209 species mixed at even abundance were detected using our matching criteria. As expected, most species received average sequence coverage around 100X, but those with closely related taxa (congeneric species or subspecies) present in the reference exceeded this value: 2 subspecies of the butterfly *Sasakia charonda* and 4 closely related species from the fly genus *Bactrocera*. Because the sequence divergences between these closely related taxa were so small (≤ 0.5%), the simulated shotgun sequences could not be unambiguously assigned to the corresponding reference barcodes, but were aligned to multiple templates.

Simulations 1B and 1C represented a more realistic scenario where a range of taxonomic groups from the bulk sample were compared against a reference barcode library, which contained a number of closely related taxa (congeners) of the targeted species. As a result, all 5 taxa were correctly identified to a species level at 100X sequencing depth. Specifically, Illumina reads were able to distinguish *Solenopsis richteri*, *Reticultitermes santonensis*, *Drosophila simulans*, and *Bactrocera papayae* from the corresponding species of close phylogenetic relatedness, using our matching criteria (> 90% coverage). The latter 3 species all had congeners in the reference that possessed < 1% interspecific divergences in their full-length barcodes. Although short reads belonging to *Sasakia charonda* were matched to both of its subspecies (*S*. *charonda* and *S*. *c*. *kuriyamaensis*), the species-level identification was achieved. The coverage of *Bactrocera* *papaya* reads to *B*. *philippinensis* was very close to our threshold (87%), and could represent a difficult example if other parameters were changed (e.g., sequencing depth, presence of closely related species in the bulk sample, variation in abundance).

The increased sequencing depth at 1,000X did not improve taxonomic resolution for the problematic taxa, specifically *Bactrocera* *papaya*. To the contrary, unambiguity in the reference-based identification method was elevated – shotgun reads belonging to *B*. *papaya* were matched to both itself and *B*. *philippinensis* at 100% coverage, while coverages to *B*. *dorsalis* and *B*. *carambolae* were raised to 84% and 83%, respectively (compared to 74% and 54% at 100X sequencing depth, respectively). All the other 4 species were successfully identified at the species level at 1,000X (Figure S7 in Additional file 3).

1. **Reference independent method**

The 15 species from simulation 2A contained 1 representative from each of the genera that had multiple species present in the reference. But no closely related species were included in this mock-up bulk sample. All 15 species were successfully *de novo* assembled into full-length barcodes. And all assemblies showed 100% identify to the corresponding reference sequence.

Simulation 2B included 4 *Drosophila* species at even abundance, 3 of which had interspecific barcode divergences < 2% (*D*. *mauritiana*, *D*. *sechellia*, and *D*. *simulans*). This mock-up sample represented an informatics challenge to the assembly approach, although such community would be rare in natural conditions. At 10X sequencing depth, we were able to assemble *COI* barcodes for *D*. *mauritiana* (553 bp) and the fourth species *D*. *melanogaster* (658 bp). However, only partial *COI* barcode (a maximum of 360 bp) was assembled for *D. simulans* due to its close relatedness to *D*. *sechellia*, whose assembly was not successful.

When closely related species co-occurred in the bulk sample, the *de novo* assembly approach seemed to favor those with higher abundance. For example, in simulation 2C, the assembling processes for both abundant species (*D*. *mauritiana* and *D*. *simulans*) produced *COI* fragments of 553 bp, while that for the 2 species with lower abundance had failed.

**Conclusions**

1. Taxonomic resolution is largely dependent on that of the full-length barcodes and is not limited by the length of Illumina shotgun reads or informatics.
2. The availability of a reference DNA barcode library will facilitate taxonomic identification at the species level. But taxon richness analysis can still be achieved without the reference library by using MOTUs as proxies, which can be *de novo* assembled and annotated from Illumina reads.
3. Both the reference-based and reference independent methods can reveal species richness information in high fidelity. But co-occurring taxa of extremely close relatedness (e.g., interspecific divergence < 1%) are difficult to distinguish, which can be further complicated when the relevant species show significantly different abundances.

**References**

68. Hu X, Yuan J, Shi Y, Lu J, Liu B, Li Z, Chen Y, Mu D, Zhang H, Li N: **pIRS: Profile-based Illumina pair-end reads simulator.** *Bioinformatics* 2012, **28**:1533–1535.

**Supplementary Tables**

**Table S3 Taxonomic, abundance, and sequencing information of simulation 1A (reference-based)**

| GI number | Order | Family | Species |
| --- | --- | --- | --- |
| 218157371 | Archaeognatha | Machilidae | *Pedetontus silvestrii* |
| 171473585 | Archaeognatha | Machilidae | *Trigoniophthalmus alternatus* |
| 62161388 | Archaeognatha | Meinertellidae | *Nesomachilis australica* |
| 242624230 | Blattaria | Blattellidae | *Blattella germanica* |
| 50812103 | Blattaria | Blattidae | *Periplaneta fuliginosa* |
| 299827802 | Blattaria | Polyphagidae | *Eupolyphaga sinensis* |
| 224587990 | Coleoptera | Aspidytidae | *Aspidytes niobe* |
| 270267691 | Coleoptera | Bostrichidae | *Apatides fortis* |
| 270267663 | Coleoptera | Buprestidae | *Acmaeodera* sp. NCS-2009 |
| 238866876 | Coleoptera | Buprestidae | *Chrysochroa fulgidissima* |
| 270267607 | Coleoptera | Cantharidae | *Chauliognathus opacus* |
| 255506410 | Coleoptera | Cerambycidae | *Psacothea hilaris* |
| 18390112 | Coleoptera | Chrysomelidae | *Crioceris duodecimpunctata* |
| 159159429 | Coleoptera | Elateridae | *Pyrophorus divergens* |
| 258649546 | Coleoptera | Gyrinidae | *Macrogyrus oblongus* |
| 21326209 | Coleoptera | Lampyridae | *Pyrocoelia rufa* |
| 270267635 | Coleoptera | Lucanidae | *Lucanus mazama* |
| 208433946 | Coleoptera | Melyridae | *Chaetosoma scaritides* |
| 258649600 | Coleoptera | Mordellidae | *Mordella atrata* |
| 208433960 | Coleoptera | Ommatidae | *Tetraphalerus bruchi* |
| 190349565 | Coleoptera | Phengodidae | *Rhagophthalmus lufengensis* |
| 192293811 | Coleoptera | Phengodidae | *Rhagophthalmus ohbai* |
| 258650138 | Coleoptera | Scarabaeidae | *Rhopaea magnicornis* |
| 208433918 | Coleoptera | Scirtidae | *Cyphon* sp. BT0012 |
| 270267294 | Coleoptera | Tenebrionidae | *Adelium* sp. NCS-2009 |
| 133755325 | Coleoptera | Tenebrionidae | *Tribolium castaneum* |
| 208433759 | Coleoptera | Trachypachidae | *Trachypachus holmbergi* |
| 13435200 | Diptera | Calliphoridae | *Chrysomya putoria* |
| 12711796 | Diptera | Calliphoridae | *Cochliomyia hominivorax* |
| 154800398 | Diptera | Calliphoridae | *Lucilia sericata* |
| 255506277 | Diptera | Cecidomyiidae | *Mayetiola destructor* |
| 255506236 | Diptera | Cecidomyiidae | *Rhopalomyia pomum* |
| 157326160 | Diptera | Ceratopogonidae | *Culicoides arakawae* |
| 164523399 | Diptera | Culicidae | *Aedes aegypti* |
| 58372083 | Diptera | Culicidae | *Aedes albopictus* |
| 299828908 | Diptera | Culicidae | *Anopheles darlingi* |
| 5834911 | Diptera | Culicidae | *Anopheles gambiae* |
| 5835918 | Diptera | Culicidae | *Anopheles quadrimaculatus* A |
| 308745776 | Diptera | Culicidae | *Culex quinquefasciatus* |
| 215259920 | Diptera | Drosophilidae | *Drosophila littoralis* |
| 45332696 | Diptera | Drosophilidae | *Drosophila mauritiana* |
| 5835233 | Diptera | Drosophilidae | *Drosophila melanogaster* |
| 45332682 | Diptera | Drosophilidae | *Drosophila sechellia* |
| 45332829 | Diptera | Drosophilidae | *Drosophila simulans* |
| 5834829 | Diptera | Drosophilidae | *Drosophila yakuba* |
| 67009990 | Diptera | Muscidae | *Haematobia irritans* |
| 120944055 | Diptera | Nemestrinidae | *Trichophthalma punctata* |
| 54306052 | Diptera | Oestridae | *Dermatobia hominis* |
| 290967645 | Diptera | Oestridae | *Hypoderma lineatum* |
| 120944038 | Diptera | Syrphidae | *Simosyrphus grandicornis* |
| 120944077 | Diptera | Tabanidae | *Cydistomyia duplonotata* |
| 312233479 | Diptera | Tachinidae | *Exorista sorbillans* |
| 156765981 | Diptera | Tephritidae | *Bactrocera carambolae* |
| 120586710 | Diptera | Tephritidae | *Bactrocera dorsalis* |
| 302632666 | Diptera | Tephritidae | *Bactrocera minax* |
| 41057423 | Diptera | Tephritidae | *Bactrocera oleae* |
| 156765967 | Diptera | Tephritidae | *Bactrocera papayae* |
| 156765995 | Diptera | Tephritidae | *Bactrocera philippinensis* |
| 309259980 | Diptera | Tephritidae | *Bactrocera tryoni* |
| 5835876 | Diptera | Tephritidae | *Ceratitis capitata* |
| 229324789 | Ephemeroptera | Ephemeridae | *Ephemera orientalis* |
| 209427686 | Ephemeroptera | Heptageniidae | *Parafronurus youi* |
| 288903297 | Ephemeroptera | Siphlonuridae | *Siphlonurus immanis* |
| 51830142 | Hemiptera | Aleyrodidae | *Aleurochiton aceris* |
| 49146478 | Hemiptera | Aleyrodidae | *Aleurodicus dugesii* |
| 52220940 | Hemiptera | Aleyrodidae | *Bemisia tabaci* |
| 51830183 | Hemiptera | Aleyrodidae | *Neomaskellia andropogonis* |
| 52221066 | Hemiptera | Aleyrodidae | *Tetraleurodes acaciae* |
| 52220968 | Hemiptera | Aleyrodidae | *Trialeurodes vaporariorum* |
| 225697513 | Hemiptera | Alydidae | *Riptortus pedestris* |
| 225676739 | Hemiptera | Anthocoridae | *Orius niger* |
| 213948225 | Hemiptera | Aphididae | *Acyrthosiphon pisum* |
| 51830049 | Hemiptera | Aphididae | *Schizaphis graminum* |
| 49146630 | Hemiptera | Aphrophoridae | *Philaenus spumarius* |
| 225697511 | Hemiptera | Aradidae | *Neuroctenus parus* |
| 225697515 | Hemiptera | Berytidae | *Yemmalysus parallelus* |
| 62161309 | Hemiptera | Cicadellidae | *Homalodisca vitripennis* |
| 225697512 | Hemiptera | Colobathristidae | *Phaenacantha marcida* |
| 225697508 | Hemiptera | Coreidae | *Hydaropsis longirostris* |
| 225697509 | Hemiptera | Cydnidae | *Macroscytus subaeneus* |
| 283099275 | Hemiptera | Delphacidae | *Laodelphax striatellus* |
| 228015376 | Hemiptera | Flatidae | *Geisha distinctissima* |
| 240266556 | Hemiptera | Fulgoridae | *Lycorma delicatula* |
| 240266730 | Hemiptera | Gelastocoridae | *Nerthra* sp. NKMT022 |
| 240266626 | Hemiptera | Gerridae | *Gerris* sp. NKMT033 |
| 240266640 | Hemiptera | Hydrometridae | *Hydrometra* sp. NKMT020 |
| 299829047 | Hemiptera | Issidae | *Sivaloka damnosus* |
| 225676740 | Hemiptera | Largidae | *Physopelta gutta* |
| 225676457 | Hemiptera | Lygaeidae | *Geocoris pallidipennis* |
| 225697510 | Hemiptera | Malcidae | *Malcus inconspicuus* |
| 240266682 | Hemiptera | Naucoridae | *Ilyocoris cimicoides* |
| 240266354 | Hemiptera | Nepidae | *Laccotrephes robustus* |
| 240266368 | Hemiptera | Notonectidae | *Enithares tibialis* |
| 240266382 | Hemiptera | Ochteridae | *Ochterus marginatus* |
| 260150943 | Hemiptera | Pentatomidae | *Halyomorpha halys* |
| 218456815 | Hemiptera | Pentatomidae | *Nezara viridula* |
| 225697506 | Hemiptera | Plataspidae | *Coptosoma bifaria* |
| 240266410 | Hemiptera | Pleidae | *Paraplea frontalis* |
| 225660332 | Hemiptera | Pyrrhocoridae | *Dysdercus cingulatus* |
| 11182462 | Hemiptera | Reduviidae | *Triatoma dimidiata* |
| 240266424 | Hemiptera | Reduviidae | *Valentia hoffmanni* |
| 225697457 | Hemiptera | Rhopalidae | *Aeschyntelus notatus* |
| 299829158 | Hymenoptera | Apidae | *Apis cerana* |
| 5834925 | Hymenoptera | Apidae | *Apis mellifera* |
| 221143400 | Hymenoptera | Apidae | *Bombus hypocrita* |
| 190349384 | Hymenoptera | Apidae | *Bombus ignitus* |
| 27733916 | Hymenoptera | Apidae | *Melipona bicolor* |
| 299827774 | Hymenoptera | Braconidae | *Cotesia vestalis* |
| 299828950 | Hymenoptera | Braconidae | *Spathius agrili* |
| 237515443 | Hymenoptera | Cephidae | *Cephus cinctus* |
| 258649392 | Hymenoptera | Evaniidae | *Evania appendigaster* |
| 312233080 | Hymenoptera | Formicidae | *Solenopsis geminata* |
| 312233122 | Hymenoptera | Formicidae | *Solenopsis invicta* |
| 312233150 | Hymenoptera | Formicidae | *Solenopsis richteri* |
| 237869070 | Hymenoptera | Ichneumonidae | *Diadegma semiclausum* |
| 306960063 | Hymenoptera | Mutillidae | *Radoszkowskius oculata* |
| 237515457 | Hymenoptera | Orussidae | *Orussus occidentalis* |
| 211908570 | Hymenoptera | Vespidae | *Abispa ephippium* |
| 148368751 | Isoptera | Rhinotermitidae | *Reticulitermes flavipes* |
| 148368779 | Isoptera | Rhinotermitidae | *Reticulitermes hageni* |
| 148368807 | Isoptera | Rhinotermitidae | *Reticulitermes santonensis* |
| 148368765 | Isoptera | Rhinotermitidae | *Reticulitermes virginicus* |
| 295065650 | Lepidoptera | Arctiidae | *Hyphantria cunea* |
| 18644896 | Lepidoptera | Bombycidae | *Bombyx mandarina* |
| 8572562 | Lepidoptera | Bombycidae | *Bombyx mori* |
| 260150957 | Lepidoptera | Crambidae | *Diatraea saccharalis* |
| 18314290 | Lepidoptera | Crambidae | *Ostrinia nubilalis* |
| 170787328 | Lepidoptera | Geometridae | *Phthonandria atrilineata* |
| 94490710 | Lepidoptera | Lycaenidae | *Coreana raphaelis* |
| 242610072 | Lepidoptera | Lymantriidae | *Lymantria dispar* |
| 312233066 | Lepidoptera | Noctuidae | *Helicoverpa armigera* |
| 195954010 | Lepidoptera | Notodontidae | *Ochrogaster lunifer* |
| 280978083 | Lepidoptera | Nymphalidae | *Acraea issoria* |
| 308745928 | Lepidoptera | Nymphalidae | *Hipparchia autonoe* |
| 297572359 | Lepidoptera | Nymphalidae | *Sasakia charonda* |
| 297572345 | Lepidoptera | Nymphalidae | *Sasakia charonda* |
| 295065636 | Lepidoptera | Papilionidae | *Papilio maraho* |
| 295065608 | Lepidoptera | Papilionidae | *Parnassius bremeri* |
| 302632610 | Lepidoptera | Papilionidae | *Teinopalpus aureus* |
| 177807247 | Lepidoptera | Pieridae | *Artogeia melete* |
| 162279939 | Lepidoptera | Saturniidae | *Antheraea pernyi* |
| 238694149 | Lepidoptera | Saturniidae | *Antheraea yamamai* |
| 238563960 | Lepidoptera | Saturniidae | *Eriogyna pyretorum* |
| 184202694 | Lepidoptera | Saturniidae | *Saturnia boisduvalii* |
| 165932395 | Lepidoptera | Sphingidae | *Manduca sexta* |
| 299829144 | Lepidoptera | Tortricidae | *Spilonota lechriaspis* |
| 84488776 | Mantodea | Mantidae | *Tamolanica tamolana* |
| 84488734 | Mantophasmatodea | Mantophasmatidae | *Sclerophasma paresisense* |
| 256985280 | Mecoptera | Panorpidae | *Neopanorpa pulchra* |
| 205351318 | Megaloptera | Corydalidae | *Corydalus cornutus* |
| 211998787 | Megaloptera | Corydalidae | *Protohermes concolorus* |
| 258649614 | Megaloptera | Sialidae | *Sialis hamata* |
| 205351332 | Neuroptera | Ascalaphidae | *Ascaloptynx appendiculatus* |
| 258649628 | Neuroptera | Mantispidae | *Ditaxis biseriata* |
| 205351290 | Neuroptera | Polystoechotidae | *Polystoechotes punctatus* |
| 306960177 | Odonata | Euphaeidae | *Euphaea formosa* |
| 229317937 | Odonata | Gomphidae | *Davidius lunatus* |
| 207268051 | Orthoptera | Acrididae | *Acrida willemsei* |
| 288900664 | Orthoptera | Acrididae | *Arcyptera coreana* |
| 207269079 | Orthoptera | Acrididae | *Calliptamus italicus* |
| 194871823 | Orthoptera | Acrididae | *Chorthippus chinensis* |
| 304322824 | Orthoptera | Acrididae | *Euchorthippus fusigeniculatus* |
| 195661170 | Orthoptera | Acrididae | *Gastrimargus marmoratus* |
| 301353420 | Orthoptera | Acrididae | *Gomphocerippus rufus* |
| 288904176 | Orthoptera | Acrididae | *Gomphocerus licenti* |
| 195933665 | Orthoptera | Acrididae | *Locusta migratoria* |
| 5835247 | Orthoptera | Acrididae | *Locusta migratoria* |
| 195661156 | Orthoptera | Acrididae | *Oedaleus decorus* |
| 283098215 | Orthoptera | Acrididae | *Ognevia longipennis* |
| 164420921 | Orthoptera | Acrididae | *Oxya chinensis* |
| 219524285 | Orthoptera | Acrididae | *Phlaeoba albonema* |
| 288903464 | Orthoptera | Acrididae | *Prumna arctica* |
| 258649420 | Orthoptera | Acrididae | *Schistocerca gregaria* |
| 288903338 | Orthoptera | Acrididae | *Traulia szetschuanensis* |
| 207268065 | Orthoptera | Gryllidae | *Myrmecophilus manni* |
| 219524229 | Orthoptera | Gryllidae | *Teleogryllus emma* |
| 58045502 | Orthoptera | Gryllotalpidae | *Gryllotalpa orientalis* |
| 207269746 | Orthoptera | Gryllotalpidae | *Gryllotalpa pluvialis* |
| 309259966 | Orthoptera | Pamphagidae | *Thrinchus schrenkii* |
| 306960149 | Orthoptera | Pneumoridae | *Physemacris variolosa* |
| 219524243 | Orthoptera | Pyrgomorphidae | *Atractomorpha sinensis* |
| 304322838 | Orthoptera | Pyrgomorphidae | *Mekongiana xiangchengensis* |
| 304322852 | Orthoptera | Pyrgomorphidae | *Mekongiella xizangensis* |
| 207270760 | Orthoptera | Rhaphidophoridae | *Troglophilus neglectus* |
| 306960135 | Orthoptera | Romaleidae | *Xyleus modestus* |
| 159524429 | Orthoptera | Tettigoniidae | *Anabrus simplex* |
| 219524090 | Orthoptera | Tettigoniidae | *Deracantha onos* |
| 299829075 | Orthoptera | Tettigoniidae | *Elimaea cheni* |
| 197935811 | Orthoptera | Tettigoniidae | *Gampsocleis gratiosa* |
| 157786563 | Orthoptera | Tettigoniidae | *Ruspolia dubia* |
| 306960090 | Orthoptera | Tridactylidae | *Ellipes minuta* |
| 312233136 | Phasmatodea | Diapheromeridae | *Micadina phluctainoides* |
| 312233193 | Phasmatodea | Heteropterygidae | *Heteropteryx dilatata* |
| 313247873 | Phasmatodea | Phasmatidae | *Entoria okinawaensis* |
| 312233307 | Phasmatodea | Phasmatidae | *Megacrania alpheus* |
| 312233164 | Phasmatodea | Phasmatidae | *Phobaeticus serratipes* |
| 313199763 | Phasmatodea | Phasmatidae | *Phraortes illepidus* |
| 312233493 | Phasmatodea | Phasmatidae | *Phraortes* sp. Iriomote Islan*d* |
| 256985350 | Phasmatodea | Phasmatidae | *Ramulus hainanense* |
| 12383036 | Phthiraptera | Boopidae | *Heterodoxus macropus* |
| 160425216 | Phthiraptera | Philopteridae | *Bothriometopus macrocnemis* |
| 89257204 | Phthiraptera | Philopteridae | *Campanulotes bidentatus* |
| 51101192 | Plecoptera | Pteronarcyidae | *Pteronarcys princeps* |
| 31324905 | Psocoptera | Lepidopsocidae | *Lepidopsocidae* sp. RS-2001 |
| 258649574 | Raphidioptera | Raphidiidae | *Mongoloraphidia harmandi* |
| 25057389 | Thysanoptera | Thripidae | *Thrips imaginis* |
| 197935825 | Thysanura | Nicoletiidae | *Atelura formicaria* |
| 42632243 | Thysanura | Lepidotrichidae | *Tricholepidion gertschi* |
| 50812159 | Thysanura | Lepismatidae | *Thermobia domestica* |

A set of 209 complete insect mitochondrial genomes was obtained from the GenBank. All MT genomes were used in simulation 1A, where all species were given even abundance and were sequenced at 100X.

**Table S4 Primer set used to pick out reference barcode region**

| **Primer** | **Sequence (5’ to 3’)** |
| --- | --- |
| LCO1490A-F | TCTCTACCAACCACAAAGACATTGG |
| HCO2198A-R | TAAACTTCNGGGTGNCCAAAGAATCA |

**Table S5 Different simulation strategies of *in silico* analysis**

| **Method** | **Reference based** | | | **Reference independent** | | |
| --- | --- | --- | --- | --- | --- | --- |
| Simulation | 1A | 1B | 1C | 2A | 2B | 2C |
| Number of species | 209 | 5 | 5 | 15 | 4 | 4 |
| Taxonomic, abundance &  sequencing information | Table S3 | Table S6 | Table S7 | Table S8 | Table S9 | Table S10 |
| Discovered species (MOTUs) | 209 | 5 | 6 | 15 | 3 | 2 |

**Table S6 Taxonomic, abundance, and sequencing information of simulation 1B (reference-based)**

| **Taxon** | **Abundance** | **Sequencing depth (X)** |
| --- | --- | --- |
| *Solenopsis richteri* | 1 | 100 |
| *Reticulitermes santonensis* | 1 | 100 |
| *Sasakia charonda* | 1 | 100 |
| *Drosophila simulans* | 1 | 100 |
| *Bactrocera papaya* | 1 | 100 |

**Table S7 Taxonomic, abundance, and sequencing information of simulation 1C (reference-based)**

| **Taxon** | **Abundance** | **Sequencing depth (X)** |
| --- | --- | --- |
| *Solenopsis richteri* | 1 | 1000 |
| *Reticulitermes santonensis* | 1 | 1000 |
| *Sasakia charonda* | 1 | 1000 |
| *Drosophila simulans* | 1 | 1000 |
| *Bactrocera papaya* | 1 | 1000 |

**Table S8 Taxonomic, abundance, and sequencing information of simulation 2A (reference independent)**

| Taxon | Abundance | Sequencing depth (X) |
| --- | --- | --- |
| *Reticulitermes santonensis* | 5 | 100 |
| *Bombyx mandarina* | 20 | 100 |
| *Bombus ignites* | 8 | 100 |
| *Rhagophthalmus lufengensis* | 1 | 100 |
| *Locusta migratoriamigratoria* | 2 | 100 |
| *Antheraea yamamai* | 5 | 100 |
| *Sasakia charonda* | 3 | 100 |
| *Anopheles darlingi* | 5 | 100 |
| *Apis cerana* | 5 | 100 |
| *Bactrocera tryoni* | 5 | 100 |
| *Solenopsis richteri* | 17 | 100 |
| *Phraortes* sp. Iriomote Island | 2 | 100 |
| *Gryllotalpa orientalis* | 7 | 100 |
| *Drosophila melanogaster* | 12 | 100 |
| *Aedes albopictus* | 16 | 100 |

**Table S9 Taxonomic, abundance, and sequencing information of simulation 2B (reference independent)**

| **Taxon** | **Abundance even** | **Sequencing depth (X)** |
| --- | --- | --- |
| *Drosophila melanogaster* | 1 | 10 |
| *Drosophila sechellia* | 1 | 10 |
| *Drosophila simulans* | 1 | 10 |
| *Drosophila mauritiana* | 1 | 10 |

**Table S10 Taxonomic, abundance, and sequencing information of simulation 2C (reference independent)**

| **Taxon** | **Abundance uneven** | **Sequencing depth (X)** |
| --- | --- | --- |
| *Drosophila melanogaster* | 1 | 10 |
| *Drosophila sechellia* | 1 | 10 |
| *Drosophila simulans* | 10 | 10 |
| *Drosophila mauritiana* | 10 | 10 |
