# Supplementary material for: Ultra-deep sequencing enables high-fidelity recovery of biodiversity for bulk arthropod samples without PCR amplification
Source: Gigascience. 2013 Mar 27;2:4. doi: 10.1186/2047-217X-2-4 (PMC3637469; doi:10.1186/2047-217X-2-4)
Supplement: Additional file 2 — Appendix S2. Analyses of taxonomic recovery for preliminary sample. [file 2047-217X-2-4-S2.docx]

**Appendix 2. Analyses of taxonomic recovery for preliminary sample**

The preliminary test sample (89 specimens) was collected in a mountainous habitat on October 2 (22°35’38.94’’N, 114°15’54.64’’E, ~ 55 m ASL) . A total of 39 OTUs were reported from 87 individuals that were successfully Sanger sequenced for the standard *COI* barcode region. The basic information including taxonomic composition (in the form of MOTUs) and data size, was summarized in Table S11. The taxonomic (MOTUs) and sequencing information was summarized in Table S11. Specimens were preserved in 99.5% ethanol at 4°C for 1 week before sequencing. Mitochondrial enrichment, DNA extraction and sequencing library preparation followed the protocol described in the main text. The preliminary sample was sequenced at an entry-level sequencing depth (~ 2.5Gb) to estimate the percentage of mitochondrial DNA after isolation and to obtain a general understanding of the proposed protocol.

**(1) Reference-based method**

Illumina reads were aligned to 39 Sanger reference MOTUs using criteria described in the Methods. A total of 25 of these reference MOTUs had sequence coverage > 90% (Table S12), therefore were considered as real taxa. One MOTU (OTU9) had a slightly lower coverage (88%) but a relatively high sequencing depth (5.1X). Thus, this MOTU was also considered as a successful recovery, which brought the total taxonomic (MOTU) recovery rate to 67.7% (true positives). Failures in detecting the remaining taxa were likely due to their small biomass, rather than taxonomic bias. Among the missed MOTUs, all but 1 were represented by a single specimen, with sizes typically < 6mm (Table S12).

**(2) Reference independent method**

When the reference barcode library was not available, Illumina reads were *de novo* assembled into 256 scaffolds using SOAPdenovo. The assembly results were promising, with a N50 of 1,401 bp and a maximum length of 15,270 bp, which was close to a complete mitochondrial genome (Table S13). Among these 256 scaffolds, a total of 25 were found containing at least part of the standard *COI* barcode region. Two of them were confirmed as bacterial contaminates (Enterobacteriaceae, *ubiquinoloxidase* gene) by searching against the Barcode of Life Data Systems and were subsequently removed from subsequent analysis. These bacterial sequences were likely derived from insect gut contents. The average length of the remaining 23 scaffolds containing the *COI* barcode region was 2,900bp (Table S13), 21 of which were > 500bp. Half of these scaffolds expanded beyond the *COI* gene.

To understand the efficiency of the reference independent method in taxonomic recovery, we compared all *COI* genes truncated from the assembled scaffolds against the barcode reference library (built from individually barcoded specimens). All but 1 of the 23 insect *COI* sequences were successfully aligned to the corresponding reference barcodes, resulting in a 56.4% (22/39) taxonomic recovery rate (true positives). Interestingly, a novel *COI* sequence belonging to the lepidopteran family Pyralidae was also detected using the reference independent approach. This sequence, however, was not found in the reference barcode library. This novel MOTU might have come from food items of predators in the collection, or ambient DNA trapped in the bulk sample. But its definite source was unclear.

Many additional mitochondrial genes had also been successfully assembled and annotated. In fact, all 13 mitochondrial protein-coding genes and both ribosomal genes were successfully assembled for at least some species present in the bulk sample (Table S11). Assembly results for all *COI* genes and long scaffolds for a few taxa were illustrated in Figure S6 in Additional file 3. Most genes could not be assembled into the same scaffolds containing *COI* due to insufficient sequencing for the given species and the lack of references for most non-*COI* genes. These genes were not shown in Figure S6 in Additional file 3.

**(3) PCR validation**

To verify the assembly results of the reference independent method, we designed sets of primers based on the assembly results and amplified and Sanger sequenced 8 genes annotated on the scaffolds (light green bars in Figure S6 in Additional file 3). All amplicons were identical to the corresponding gene assembly. The nearly complete mitochondrial genome assembled in preliminary sample was essentially identical to that of *Halyomorpha halys* (Hemiptera: Pentatomidae) from GenBank (gi260150943), with just a few single nucleotide polymorphisms.

**Supplementary Tables**

**Table S11 Sample composition, sequencing information and *COI* recovery rates of the preliminary sample**

| Number of Individuals | 89 |
| --- | --- |
| Number of *COI* barcodes obtained | 85 |
| Number of MOTUs (2%) | 39 |
| Raw data size (Gb) | 2.5 |
| High quality data size (Gb) | 2.2 |
| Discovery rate (with reference) | 67% |
| Discovery rate (no reference) | 56% |
| Assembly coverage rate (% MT genomes) | 38% |
| Total length and percentage of *COI* genes ^1^ | 26,649 (46%) |
| Number of assembled mitochondrial genes ^2^ | 372 |

^1^ The total length (bp) of assembled *COI* genes. And the percentage of assembled *COI* genes of 37 full length *COI* genes (~1,530bp each).

^2^Note that a small portion of the genes were assembled into two scaffolds.

**Table S12 Taxonomic recovery using the reference-based and reference independent methods**

|  |  |  |  | **Taxon recovered** | |
| --- | --- | --- | --- | --- | --- |
| **Taxonomic groups** | **MOTUs** | **Number of individuals** | **Total length (mm)** | **Ref. based** | **Ref. independent** |
| Diptera | OTU8 | 1 | 1.5 |  |  |
|  | OTU14 | 1 | 5 | ● |  |
|  | OTU15 | 1 | 3 |  |  |
|  | OTU17 | 5 | 17 |  |  |
|  | OTU21 | 1 | 2 |  |  |
|  | OTU25 | 1 | 4 |  |  |
|  | OTU34 | 1 | 6 | ● | ● |
| Hemiptera | OTU19 | 1 | 6 | ● | ● |
|  | OTU22 | 1 | 7 | ● | ● |
|  | OTU28 | 1 | 6 |  |  |
|  | OTU29 | 1 | 18 | ● | ● |
| Hymenoptera | OTU18 | 1 | 3 |  |  |
|  | OTU37 | 1 | 5 |  |  |
| Lepidoptera | OTU1 | 2 | 27 | ● | ● |
|  | OTU2 | 2 | 9 | ● |  |
|  | OTU3 | 13 | 66 | ● | ● |
|  | OTU4 | 1 | 5 |  |  |
|  | OTU5 | 8 | 43 | ● |  |
|  | OTU6 | 7 | 130 | ● | ● |
|  | OTU7 | 3 | 28 | ● | ● |
|  | OTU9 | 1 | 6 | ● | ● |
|  | OTU10 | 1 | 3 |  |  |
|  | OTU11 | 4 | 54 | ● | ● |
|  | OTU12 | 1 | 7 | ● | ● |
|  | OTU13 | 9 | 61 | ● | ● |
|  | OTU16 | 1 | 23 | ● | ● |
|  | OTU20 | 1 | 13 | ● | ● |
|  | OTU23 | 1 | 4 |  |  |
|  | OTU24 | 1 | 4 |  |  |
|  | OTU27 | 1 | 16 | ● | ● |
|  | OTU30 | 1 | 11 | ● | ● |
|  | OTU31 | 2 | 10 | ● | ● |
|  | OTU32 | 2 | 14 | ● | ● |
|  | OTU33 | 1 | 7 | ● | ● |
|  | OTU35 | 1 | 5 | ● |  |
|  | OTU36 | 1 | 6 |  |  |
|  | OTU38 | 1 | 11 | ● | ● |
|  | OTU39 | 1 | 13 | ● | ● |
| Psocoptera | OTU26 | 1 | 5 | ● | ● |
| Novel MOTUs | Novel2 |  |  |  | ● |
| Total | 40 | 85 |  | 26 | 23 |

* A black dot indicates the existence of corresponding MOTU

**Table S13 Results of *de novo* assembly for preliminary sample**

|  | Length (bp) | |
| --- | --- | --- |
|  | *COI* scaffolds | Mitochondrial scaffolds |
| Minimum | 406 | 106 |
| Average | 2,900 | 900 |
| N50 | 5,519 | 1,401 |
| N90 | 1,380 | 367 |
| Maximum | 15,270 | 15,270 |
