# Supplementary material for: Ultra-deep sequencing enables high-fidelity recovery of biodiversity for bulk arthropod samples without PCR amplification
Source: Gigascience. 2013 Mar 27;2:4. doi: 10.1186/2047-217X-2-4 (PMC3637469; doi:10.1186/2047-217X-2-4)

**Figure S1. Taxonomic composition of bulk arthropod Preliminary samples & Formal sample.** A Neighbor-Joining tree of COI barcode sequences extracted from individual specimens. The NJ tree was constructed using MEGA 5.0 using a distance method and default parameters. Tree terminals were collapsed into triangles using tools provided by the Interactive Tree of Life using an arbitrary threshold of 2%, as a rough estimation for the species diversity. MOTUs found in preliminary study and the formal sample are marked in red and blue colors, respectively. Four species found in both samples were marked in green color.

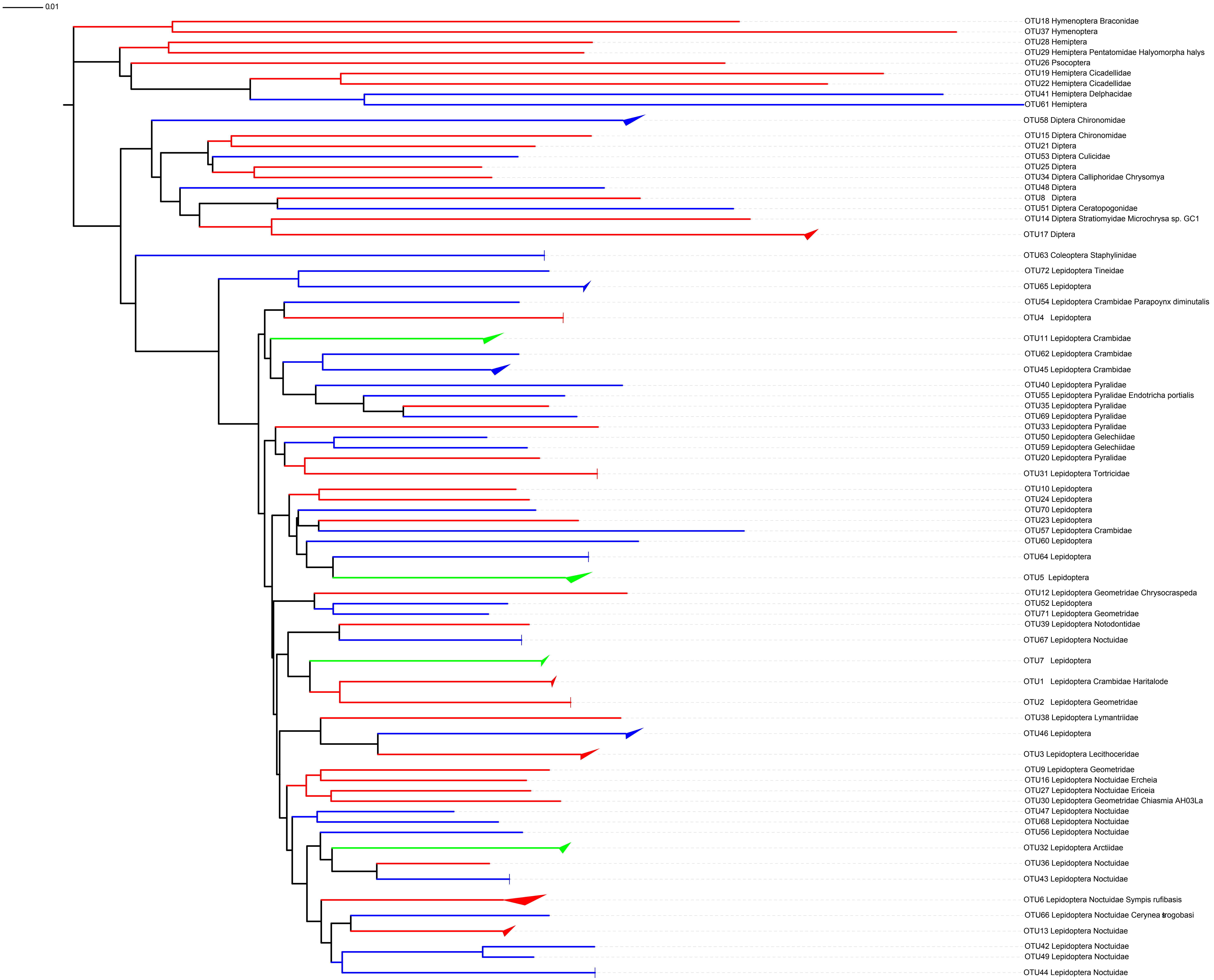

**Figure S2: The schematic demonstration of the matching criteria employed in the reference-based method.** Only when the coverage of a reference by Illumina reads is evenly distributed, as shown in Reference 1, this taxon is considered successfully detected. Specifically, >90% of the reference sequence has to be matched at >99% similarity, where the reference coverage rate

$$= \frac{\text{mapped sites on reference}}{\text{total length of reference}} .$$

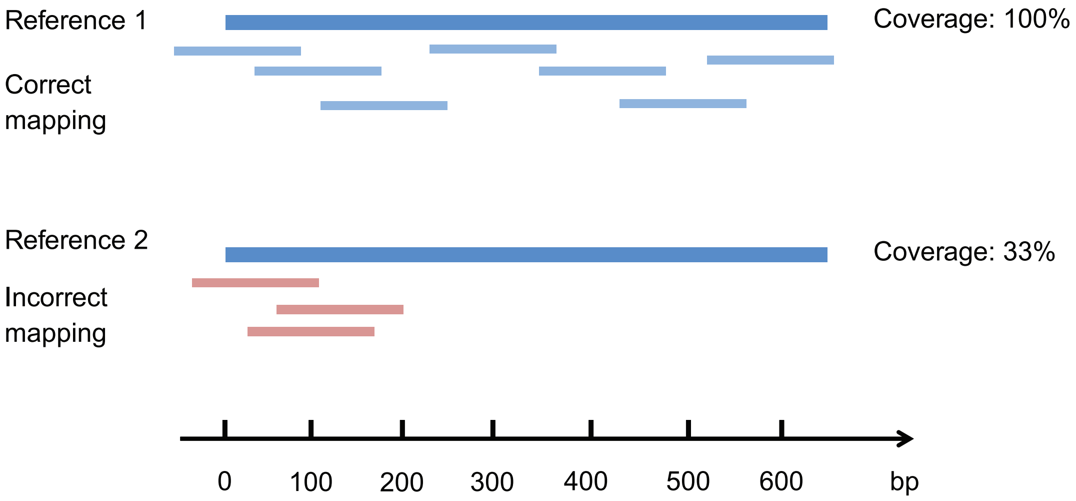

**Figure S3: Venn Diagram for the MOTU discoveries for using reference-based and reference independent methods**

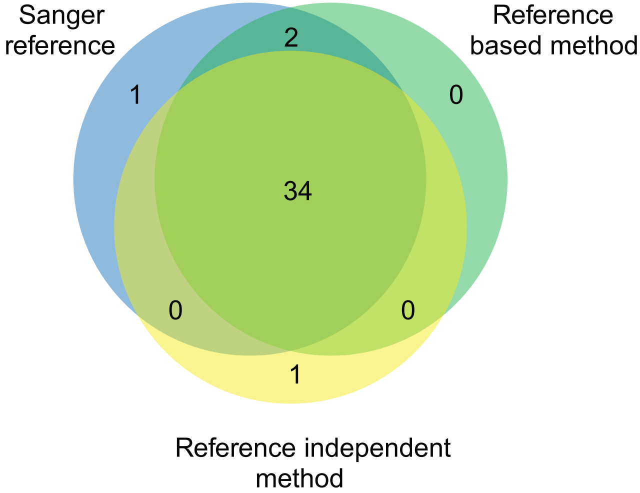

**Figure S4: Correlation between biomass and data volume with different biomass equations.** Two more biomass equations were tested. (A): biomass was calculated based on the method of Ganihar et al. [65] (B): biomass was estimated with the parameters of Hódar et al. [66] All the P values of coefficients are significant (<0.001).

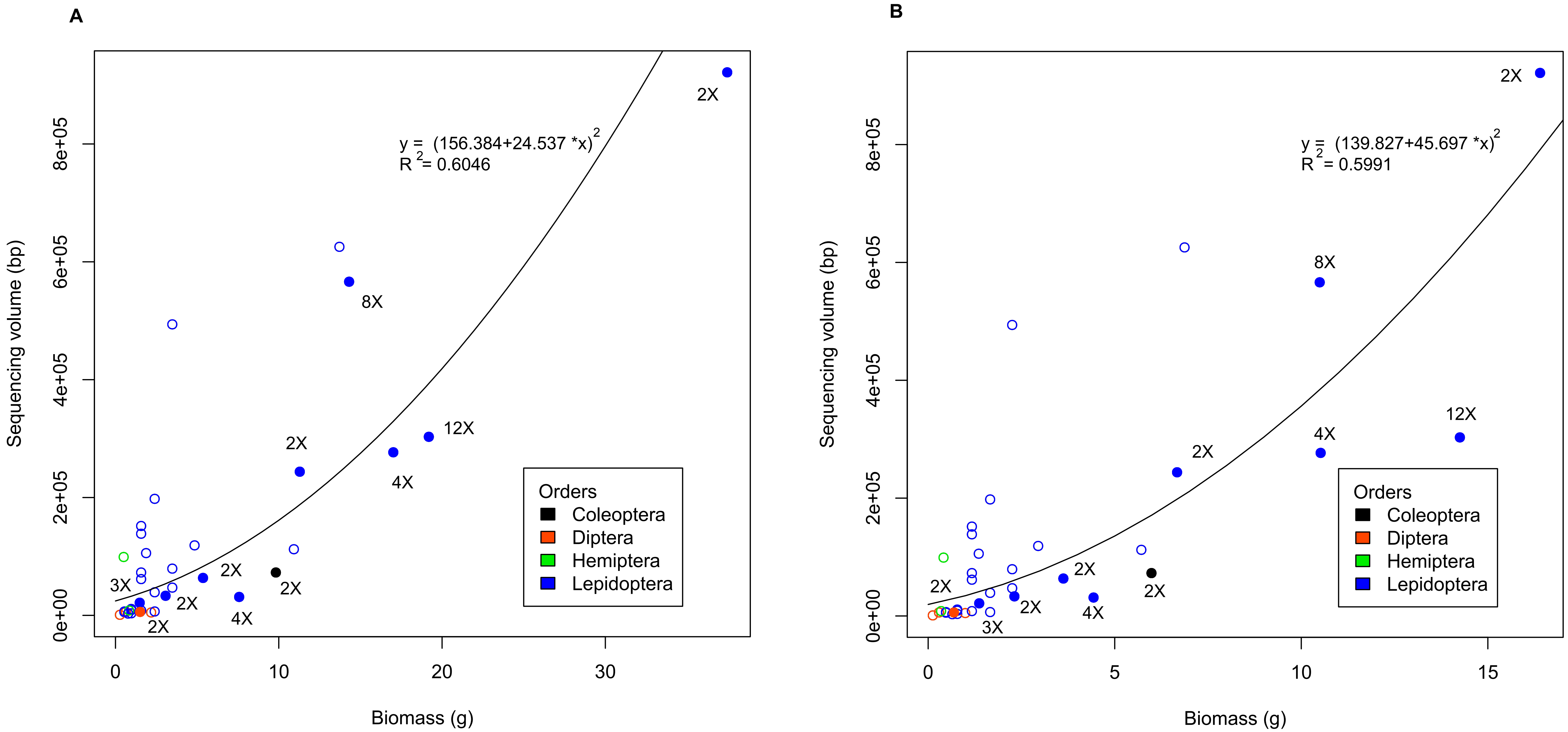

**Figure S5: Ranges of arthropod body-sizes that were detected and missed at given sequencing volumes.** Apparent divergences of body-sizes between detected and missed taxa were observed along the increase of sequencing volume in both methods. Deeper sequencing enabled detections of smaller taxa.

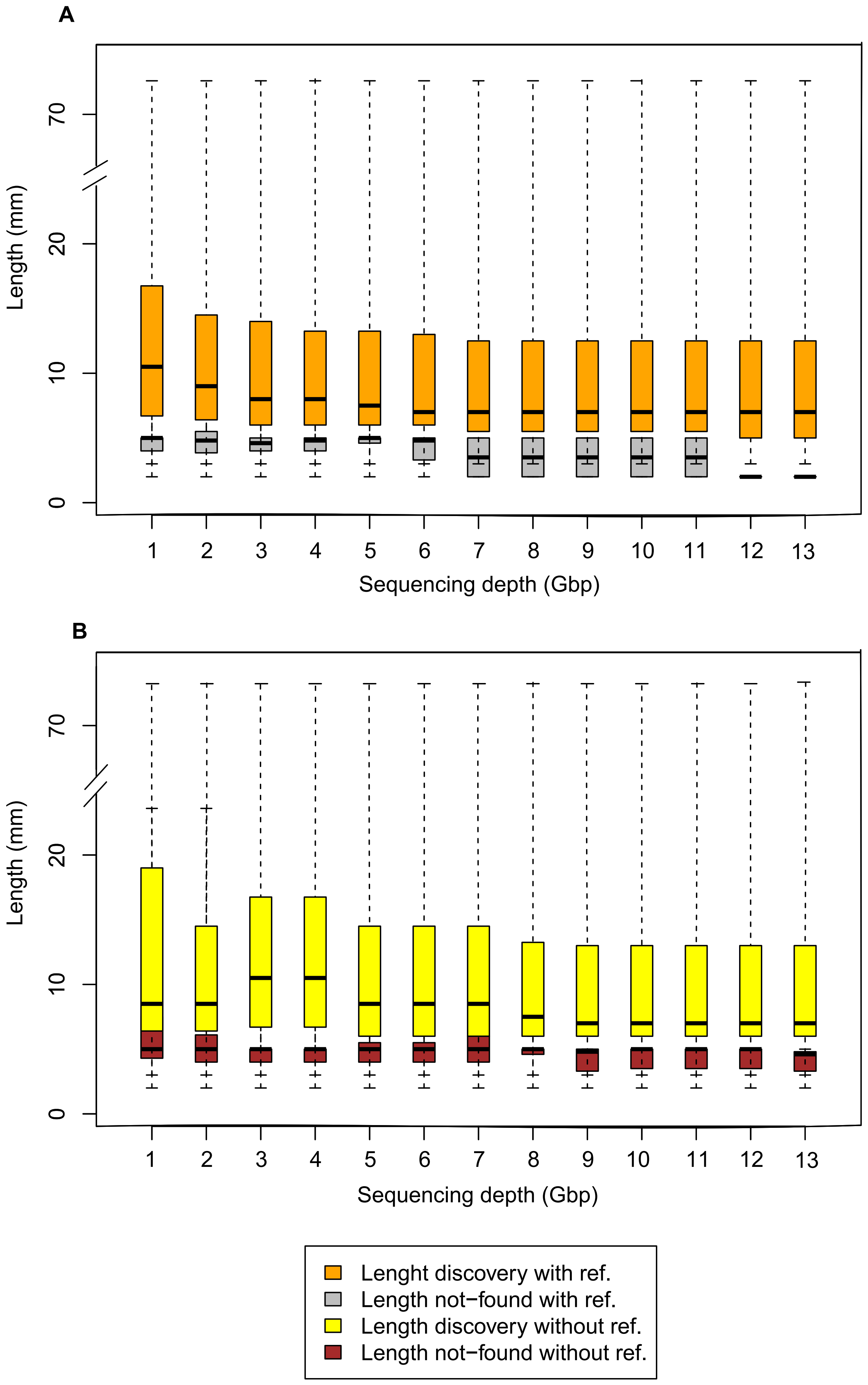

**Figure S6: Assembly results for all COI genes and long scaffolds containing additional mitochondrial genes in Preliminary sample (2.5 Gb).**
Dark green bars represent successfully annotated genes; black bars represent gaps; and light green bars represent genes confirmed by PCR validation. Non-COI genes that cannot be assembled into same scaffolds containing COI are not shown in the figure.

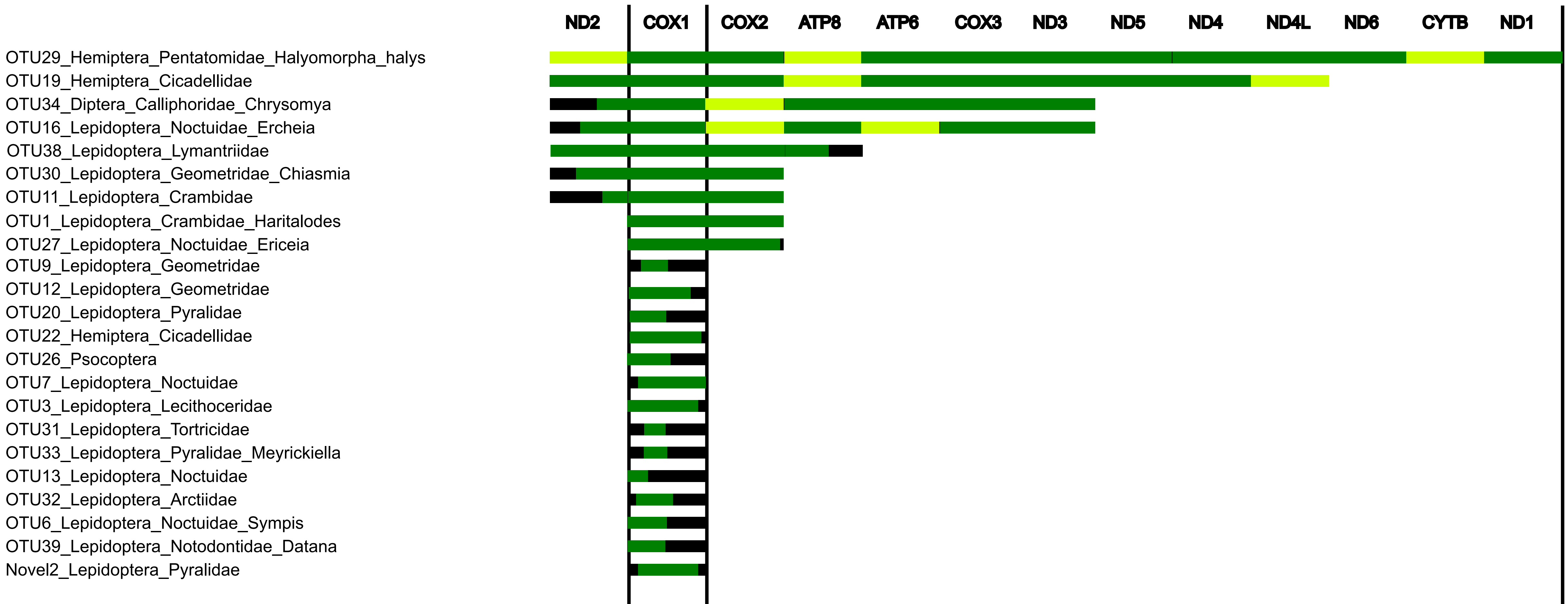

**Figure S7: Thresholds of taxonomic resolution using simulated Illumina shotgun reads.** The distance tree is built based on 24 species from 6 insect genera, each of which are represented by multiple closely related species. The red line indicates the taxonomic resolution using the proposed NGS methods at 100X sequencing depth. The gray line indicates the taxonomic resolution may decrease to about 1%, with the increase of sequencing depth.

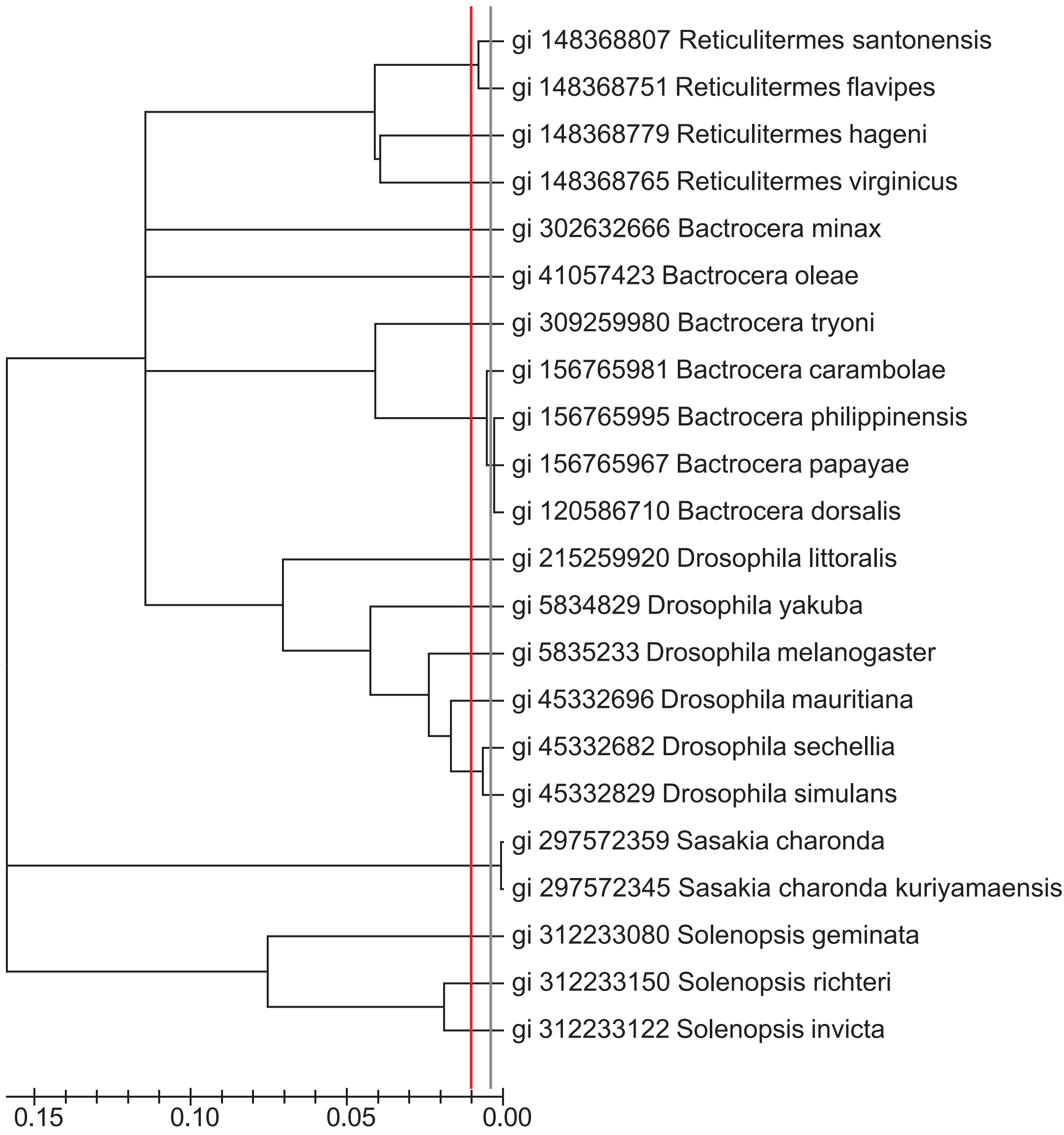

Supplement: Additional file 3: Figure S1 — Taxonomic composition of bulk arthropod Preliminary samples & Formal sample. A Neighbor-Joining tree of COI barcode sequences extracted from individual specimens. The NJ tree was constructed using MEGA 5.0 using a distance method and defaultparameters. Tree terminals were collapsed into triangles using tools provided by the Interactive Tree of Life using an arbitrary threshold of 2%, as a rough estimation for the species diversity. MOTUs found in preliminary study and the formal sample are marked in red and blue colors, respectively. Four species found in both samples were marked in green color. Figure S2. The schematic demonstration of the matching criteria employed in the reference-based method. Only when the coverage of a reference by Illumina reads is evenly distributed, as shown in Reference 1, this taxon is considered successfully detected. Specifically, >90% of the reference sequence has to be matched at >99% similarity, where the reference coverage rate. Figure S3. Venn Diagram for the MOTU discovery for using reference-based and reference independent methods. Figure S4. Correlation between biomass and data volume with different biomass equations. Two more biomass equations were tested. (A): biomass was calculated based on the method of Ganihar et al. [65] (B): biomass was estimated with the parameters of Hódar et al. [66] All the P values of coefficients are significant (<0.001). Figure S5. Ranges of arthropod body-sizes that were detected and missed at given sequencing volumes. Apparent divergences of body-sizes between detected and missed taxa were observed along the increase of sequencing volume in both methods. Deeper sequencing enabled detections of smaller taxa. Figure S6. Assembly results for all COI genes and long scaffolds containing additional mitochondrial genes in Preliminary sample (2.5 Gb). Dark green bars represent successfully annotated genes; black bars represent gaps; and light green bars represent genes confirmed by PCR validation. Non-CO [file 2047-217X-2-4-S3.pdf]
